# Structure–Activity Relationships of 7-Substituted Dimethyltyrosine-Tetrahydroisoquinoline Opioid Peptidomimetics

**DOI:** 10.3390/molecules24234302

**Published:** 2019-11-26

**Authors:** Deanna Montgomery, Jessica P. Anand, Mason A. Baber, Jack J. Twarozynski, Joshua G. Hartman, Lennon J. Delong, John R. Traynor, Henry I. Mosberg

**Affiliations:** 1 Department of Medicinal Chemistry, College of Pharmacy, University of Michigan, Ann Arbor, MI 48109, USA; dmontg@umich.edu (D.M.); mbaber@rollins.edu (M.A.B.); 2 Department of Pharmacology, Medical School, University of Michigan, Ann Arbor, MI 48109, USA; janand@umich.edu (J.P.A.); jactwaro@umich.edu (J.J.T.); hartjosh@umich.edu (J.G.H.); delongl@umich.edu (L.J.D.); jtraynor@umich.edu (J.R.T.); 3 Edward F. Domino Research Center, Medical School, University of Michigan, Ann Arbor, MI 48109, USA

**Keywords:** peptidomimetic, structure-activity, opioids, multifunctional ligands

## Abstract

The opioid receptors modulate a variety of biological functions, including pain, mood, and reward. As a result, opioid ligands are being explored as potential therapeutics for a variety of indications. Multifunctional opioid ligands, which act simultaneously at more than one type of opioid receptor, show promise for use in the treatment of addiction, pain, and other conditions. Previously, we reported the creation of bifunctional kappa opioid receptor (KOR) agonist/mu opioid receptor (MOR) partial agonist ligands from the classically delta opioid receptor (DOR) antagonist selective dimethyltyrosine-tetrahydroisoquinoline (Dmt-Tiq) scaffold through the addition of a 7-benzyl pendant on the tetrahydroisoquinoline ring. This study further explores the structure–activity relationships surrounding 7-position pendants on the Dmt-Tiq scaffold. Some analogues maintain a KOR agonist/MOR partial agonist profile, which is being explored in the development of a treatment for cocaine addiction. Others display a MOR agonist/DOR antagonist profile, which has potential to be used in the creation of a less addictive pain medication. Ultimately, we report the synthesis and in vitro evaluation of novel opioid ligands with a variety of multifunctional profiles.

## 1. Introduction

Opioids have one of the longest known histories of any drug class. The use of opium for ritual, medicinal, and/or recreational purposes dates back to ancient civilizations [1,2]. In the early 1800s, Friedrich Sertürner isolated the primary active ingredient of opium and named it morphine [1]. This began the chemical exploration of the opiates, and ultimately, led to discovery of the opioid receptors and their endogenous ligands. It is widely accepted that there are three major types of opioid receptors—the kappa opioid receptor (KOR), the mu opioid receptor (MOR), and the delta opioid receptor (DOR). These receptors have high sequence and structural homology, and they are all Class A GPCRs [3,4]. The structure and function of this type of receptor have been thoroughly reviewed [5,6]. Though most well-known for its role in regulating pain, the opioid system is also involved in many other biological processes, including mood [7,8,9] and reward [8,10]. As such, opioids remain an important and promising class of molecules for the development of therapeutics for a variety of indications.

Functions of the opioid receptors are modulated by both endogenous and exogenous opioid ligands. In the two centuries since the discovery of morphine, many semi-synthetic and synthetic opioids have been developed for this purpose. As the complex pharmacology of the opioid system continues to be revealed, it has been posited that unwanted effects and desired effects may result from the same interaction of an opioid agonist or antagonist with its target. As a result, the development of selective agents has declined, and the development of multifunctional ligands, compounds that act simultaneously at multiple opioid receptor types, has gained popularity as a strategy for the design of therapeutics [11,12]. The current state of multifunctional opioid ligands has recently been reviewed [13].

**Figure 1:**
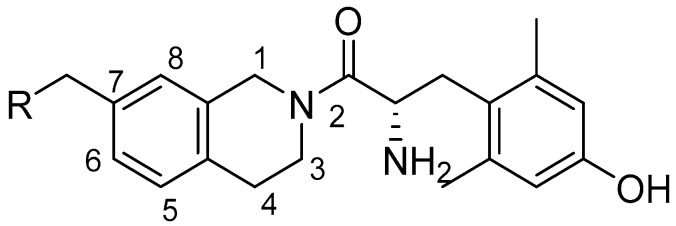
A variety of pendants were introduced at the 7-position of the tetrahydroisoquinoline of the dimethyltyrosine-tetrahydroisoquinoline (Dmt-Tiq) scaffold.

Our group [14] and others [15,16,17,18,19] have shown that the dimethyltyrosine-tetrahydroisoquinoline (Dmt-Tiq) scaffold can be used in the development of multifunctional opioid ligands (Figure 1). This scaffold, originally developed as a selective DOR antagonist, has been extensively explored through synthesis of many analogues. However, the confines of traditional peptide synthesis have limited substitution on the tetrahydroisoquinoline (Tiq) ring. Nearly two decades ago, minor substitutions were reported at the 6-, 7-, and 8- positions, but all of these compounds displayed a DOR antagonist profile similar to that of the parent peptide [17,20]. Recently, we reported that installation of a 7-benzyl pendant on the Tiq could alter the profile of this series to KOR agonism/MOR partial agonism [14].

KOR agonists have shown potential for use in the treatment of cocaine addiction because of their reward-modulating properties. Specifically, administration of a KOR agonist can reduce cocaine self-administration in non-human primates [21,22]. However, KOR agonism is also associated with dysphoria, an intense feeling of unease or dissatisfaction [7]. As a result, selective KOR agonists have limited therapeutic potential. It is well known that MOR agonism is associated with euphoria. Therefore, a bifunctional KOR agonist/MOR agonist offers a potential alternative to a selective KOR agonist that may result in a more favorable side effect profile. In fact, there is evidence to suggest that a KOR agonist/MOR agonist may be useful in the treatment of cocaine addiction [23,24].

The aim of this work was to explore structure–activity relationships around the 7-benzyl pendant which introduced KOR agonism to the Dmt-Tiq scaffold. Novel analogues reported here reveal that substitution on the benzyl ring can maintain a KOR agonist/MOR partial agonist profile while analogues with other 7-position pendants show varied results. Overall, this work demonstrates the development of novel Dmt-Tiq peptidomimetics that display a range of multifunctional opioid profiles.

## 2. Results

A series of novel Dmt-Tiq compounds with substitution at the Tiq 7-position were prepared and evaluated in vitro for opioid activity.

### 2.1. Synthesis

**Scheme 1:**
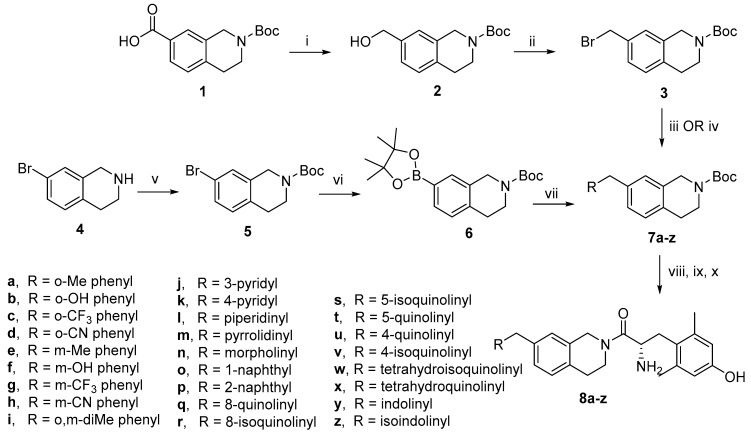
Preparation of Dmt-Tiq peptidomimetics. *Reagents and conditions*: (i) BH_3_ SMe_2_, THF; (ii) CBr_4_, PPh_3_, DCM; (iii) substituted aryl boronic acid, Pd(dppf)Cl_2_, K_2_CO_3_, 3:1 acetone:water; (iv) amine, K_2_CO_3_, DMF; (v) Boc_2_O, microwave; (vi) bis(pinacolato)diboron, Pd(dppf)Cl_2_, CH_3_CO_2_K, DMSO; (vii) substituted benzyl bromide, Pd(dppf)Cl_2_, K_2_CO_3_, 3:1 acetone/water; (viii) HCl, 1,4-dioxane or TFA, DCM; (ix) diBoc-Dmt, PyBOP, 6Cl-HOBt, DIEA, DMF; (x) TFA, DCM.

All compounds were prepared from commercial starting materials according to one of the synthetic routes shown in Scheme 1. In the first route, commercially available carboxylic acid **1** was reduced to the corresponding secondary alcohol **2** using borane dimethylsulfide. An Appel reaction was performed to convert alcohol **2** to benzyl bromide **3**. The pendant was then attached via Suzuki coupling of intermediate **3** with the corresponding boronic acid or S_N_2 reaction with the corresponding nucleophile. In the second route, Boc-protected tetrahydroisoquinoline **5** was prepared from commercially available 7-bromotetrahydroisoquinoline (**4**). This intermediate was converted to boronic ester **6**, and the appropriate pendant was attached by Suzuki coupling with the corresponding benzyl bromide. In each case, after the pendant was attached, the Boc group was removed from intermediate **7a**–**z** with acid, and the deprotected tetrahydroisoquinoline intermediate was coupled with diBoc-protected dimethyltyrosine. Finally, the Boc groups were removed to yield the final peptidomimetic **8a**–**z**.

### 2.2. Pharmacological Evaluation

Each novel compound was evaluated for binding to and stimulation of KOR, MOR, and DOR. Binding affinity (K_i_) was determined by competitive displacement of [^3^H]-diprenorphine, a non-selective opioid receptor antagonist with similar affinity for each of the three receptors. Potency (EC_50_) and efficacy, expressed as percent stimulation compared to a standard agonist at each receptor, were determined by a [^35^S]-GTPγS binding assay.

Building on our previous work [14], several ortho and meta substituents on the benzyl ring were investigated as well as the *o*-,*m*-dimethyl analogue. The results of the pharmacological evaluation of these compounds are shown in Table 1. Data for the previously reported 7-benzyl analogue **4c** is shown for comparison. Previously, this compound was evaluated at human KOR, rat MOR, and rat DOR. The profile shown here differs slightly from that previously reported because all compounds in this study were evaluated only at human receptors. All ortho- and meta-substituted analogues reported here display single digit nanomolar or subnanomolar binding at all three opioid receptors. In general, ortho analogues show the highest affinity for KOR compared to the other receptors, while most meta analogues show the highest affinity for DOR. Compared to standard agonists, each of these analogues retains moderate (54%) to high (89%) efficacy at KOR and low (29%) to high efficacy (81%) at MOR. Most analogues show no DOR agonism, but the ortho trifluoromethyl analogue **8c** shows low DOR efficacy and potency. Potency for these compounds remains primarily in the double or triple digit nanomolar range. The balance of potencies varies for ortho analogues, while meta analogues and the disubstituted analogue are consistently more potent at MOR than KOR.

Next, we explored the incorporation of nitrogen into the aromatic ring of the pendant. In place of the benzyl pendant, 3- and 4-pyridyl pendants were added at the 7-position of the tetrahydroisoquinoline ring with a methylene spacer (Table 2). Due to well-known synthetic difficulties [25], the 2-pyridyl analogue was not successfully synthesized. Evaluation of the pyridyl analogues revealed a loss in binding and a drastic loss of potency at KOR with low efficacy at MOR and no agonism at DOR, compared to their carbocyclic counterparts.

Non-aromatic pendants were also explored. Table 3 shows pharmacological data for analogues with saturated, cyclic amine pendants. For this series, all KOR agonism was lost, and only weak potency and low efficacy at MOR were observed.

To test whether opioid activity could be maintained in the presence of larger pendants, 1- and 2-naphthyl analogues were synthesized. These results are shown in Table 4. The 1-naphthyl pendant displays a KOR agonist/MOR partial agonist profile, while the 2-naphthyl pendant results in a drastic loss in KOR binding and a complete loss of KOR agonism.

Given the MOR/KOR profile of analogue **8o**, a nitrogen scan was conducted to further explore the structure–activity relationships around this pendant (Table 5). Similar to the pyridyl analogues, synthetic difficulties prevented the synthesis and evaluation of the 1-isoquinolinyl analogue. With few exceptions, single digit nanomolar or stronger binding is observed at all three receptors, and these analogues favor binding to DOR over MOR and KOR. All of these compounds show MOR agonism, and all except analogue **8u** display partial to full KOR agonism. However, only compounds **8q** and **8v** shows DOR agonism. Ultimately, the addition of a single nitrogen to this ring results in compounds that show a range of multifunctional opioid profiles.

Finally, bicyclic pendants with one saturated ring and one aromatic ring were explored. This subset of analogues displays two distinct profiles (Table 6). Compounds **8x** and **8y** show balanced affinity and efficacy at KOR and MOR, while compounds **8w** and **8z** display a loss in KOR binding and no KOR agonism. However, the latter two compounds show potent, moderate to high efficacy at MOR and strong binding but no agonism at DOR.

## 3. Discussion

Previous work by our group [14] and others [15,16,17,18,19] has indicated that the classically DOR antagonist selective dimethyltyrosine-tetrahydroisoquinoline (Dmt-Tiq) scaffold can be used as a starting point for the development of multifunctional opioid ligands. Building on our previous work, this study further explores installation of a 7-position pendant on the tetrahydroisoquinoline ring as a means of developing ligands with pharmacologically useful, multifunctional profiles. Previously, we reported that introduction of ortho and meta substituents onto a 7-benzyl pendant could produce ligands that demonstrate KOR agonism and MOR partial agonism [14], a bifunctional profile which has shown promise for the treatment of addiction to cocaine and other drugs of abuse. Here, we further explore the structure–activity relationships surrounding this novel series of opioid ligands and report compounds with this and other multifunctional opioid profiles.

Based on previously reported initial results from this series [14], we believed ortho and meta substituents on a 7-benzyl pendant to be promising structural modifications for the development of KOR agonist/MOR partial agonist ligands. A series of additional ortho and meta substitutions were evaluated to confirm whether they would exhibit the anticipated profile (Table 1). As expected, ortho and meta substitutions on the 7-benzyl pendant are favorable for the development of KOR/MOR ligands. Because di-substitution (compound **8i**) results in a notable drop in KOR potency, it shows no advantage over a single ortho or meta substituent. A few of the ligands in this series, including analogue **8c**, show DOR agonism, which represents a problem for the development of a therapeutically useful KOR/MOR ligand because DOR agonism is associated with problematic side effects, including convulsions [9,26,27]. On the other hand, DOR antagonism may be beneficial for the development of a treatment for addiction since it has been shown to lower the addiction potential of MOR agonists in preclinical models [28,29,30]. The strong MOR agonism of some compounds in this series (compounds **8c** and **8f**) is also a concern for the development of a therapy, as this activity would likely impart greater abuse potential. The most promising compound in this series for the development of a KOR agonist/MOR partial agonist for treatment of cocaine addiction, analogue **8b**, shows high potency and efficacy at KOR, high potency and low efficacy at MOR, and is devoid of DOR agonism. This compound also has higher affinity for KOR and MOR than for DOR (eight-fold and two-fold, respectively), making it a promising candidate for further evaluation.

The introduction of a nitrogen to the 7-benzyl ring was not favorable for the development of a KOR/MOR ligand (Table 2). Rather, these analogues are selective for DOR over KOR and MOR and display low potency and efficacy at KOR and MOR. Replacement of the benzyl pendant with a saturated, cyclic amine pendant likewise decreases MOR potency drastically and eliminates KOR agonism altogether (Table 3). Unlike many of the compounds reported here, these analogues do not show particularly useful opioid profiles.

To explore the potential of installing larger pendants at the Tiq 7-position, we first synthesized analogues with 1- and 2-naphthyl pendants (Table 4). Though the high clogP (5.6) and associated insolubility of these compounds is a problem for the ultimate development of a therapeutic, they were prepared as useful probes to further explore what might be tolerated in this series. Based on our previous observations from ortho, meta, and para substitutions [14], we hypothesized that the 1-naphthyl pendant would be favorable for the development of a KOR agonist while the 2-naphthyl pendant would not. The 1-naphthyl pendant points in the same direction as ortho and meta substituents, where there is room for additional steric bulk to be accommodated in the active configuration of the KOR orthosteric site. The 2-naphthyl pendant, on the other hand, points in the direction of meta and para substituents, where it clashes with the receptor. As expected, the 1-naphthyl analogue **8o** shows the desired KOR/MOR profile, while the 2-naphthyl analogue **8p** shows a drastic decrease in KOR binding and a complete loss of KOR agonism.

Based on these findings, we conducted a nitrogen scan of the 1-naphthyl pendant. The introduction of a single nitrogen drops the clogP by approximately 1.5 units, making these analogues much more promising candidates for use in animal studies and clinical settings. The profile of these analogues differed based on the placement of the nitrogen. Most of these compounds (analogues **8q**–**8v**) show some degree of KOR agonism and MOR agonism, but only analogue **8t** displays the desired KOR agonist/MOR partial agonist profile. Notably, this compound is equipotent at KOR and MOR and is a promising candidate for further study. It is approximately 3-fold selective for KOR and DOR over MOR which may lower the abuse potential of such a compound. Analogue **8u** displays partial agonism only at MOR but high affinity for DOR, a profile most similar to that of previously reported Dmt-Tiq compounds. On the other hand, compound **8v** has a potent KOR agonist/MOR agonist/DOR partial agonist profile and is weakly selective for KOR and DOR over MOR. While interesting, this profile is likely clinically irrelevant.

Finally, we explored bicyclic pendants with a cyclic amine attached to an aromatic ring. The profile differs, likely due to the placement of the second ring within the receptor binding site. As expected, those that would most closely mimic the 1-naphthyl pendant, compounds **8w** and **8z**, exhibit a KOR agonist/MOR agonist profile. The binding and efficacy profile at KOR and MOR for these two compounds is remarkably balanced, though they are more potent at MOR (five-fold and two-fold, respectively). As discussed above, the higher MOR efficacy and potency of these compounds compared to others would likely impart greater addiction potential. On the other hand, those compounds which more closely mimic the 2-naphthyl pendant, analogues **8w** and **8z**, show no KOR agonism, as expected. However, these analogues exhibit MOR agonism and DOR antagonism, a bifunctional profile being explored in the development of a less addictive treatment for pain [13,31,32]. Both compounds display potent MOR agonism and selectivity for MOR and DOR over KOR (18-fold and 43-fold, respectively). In addition, compound **8z** shows balanced affinity at MOR and DOR, a quality previously explored by our group as a way to mitigate addiction potential [33]. These compounds represent a starting point for further study for the development of a MOR agonist/DOR antagonist.

In conclusion, this work reports novel opioid ligands with a variety of multifunctional profiles. We have further elucidated structure–activity relationships surrounding the 7-position pendant on the tetrahydroisoquinoline ring of the classically DOR antagonist selective Dmt-Tiq scaffold. Ortho substituted analogues and select bicyclic pendant analogues show promise for the development of a KOR agonist/MOR partial agonist, a profile being investigated for the treatment of cocaine addiction. Compounds **8w** and **8z** exhibit a balanced MOR agonist/DOR antagonist profile and have potential to be investigated as a treatment for pain with lowered addiction potential. Future work will examine the pharmacokinetic properties of these compounds and explore the in vivo activity of interesting compounds from this series.

## 4. Materials and Methods

### 4.1. Chemistry

The chemical methods used were the same as those previously described [14] with any changes noted below. Unless otherwise noted, all reagents and solvents were purchased from commercial sources and used without additional purification. DiBoc-DMT was prepared from commercially available DMT according to standard procedures as previously reported [14]. Microwave reactions were performed in a Discover SP microwave synthesizer (CEM Corp., Matthews, NC, USA) in a closed vessel with maximum power input of 300 W. Column chromatography was carried out on silica gel cartridges using an Isolera One flash purification system (Biotage AB, Uppsala, Sweden) with a linear gradient of 100% hexanes to 100% ethyl acetate. Before chromatographic purification, crude reaction mixtures were analyzed by thin layer chromatography in hexanes/ethyl acetate. Purification of final compounds was performed using asemipreparative HPLC (Waters Technologies Corp., Milford, MA, USA) with a Vydac protein and peptide C18 reverse phase column using a linear gradient of 100% solvent A (water with 0.1% TFA) to 100% solvent B (acetonitrile with 0.1% TFA) at a rate of 1% per minute with UV absorbance monitored at 230 nm. Purity of final compounds was determined on an Alliance 2690 analytical HPLC (Waters Technologies Corp., Milford, MA, USA) with a Vydac protein and peptide C18 reverse phase column using the same gradient with UV absorbance monitored at 230 nm. Purity of final compounds used for testing was ≥95% as determined by HPLC. ^1^H-NMR data for intermediates and final compounds in CDCl_3_ or CD_3_OD was obtained on a 400 MHz or 500 MHz Varian spectrometer (Agilent Technologies Inc., Santa Clara, CA, USA). EIMS data was obtained using an Agilent 6130 HPLC-MS (Agilent Technologies Inc., Santa Clara, CA, USA) in positive ion mode. HREIMS data was obtained using an Agilent QTOF HPLC-MS (Agilent Technologies Inc., Santa Clara, CA, USA) in positive ion mode.

#### 4.1.1. General Procedure A for Microwave Suzuki Coupling of Benzyl Bromide 3 and Pendant Boronic Acid

Benzyl bromide **3** (1.0 eq), the appropriate boronic acid (1.5 eq), Pd(dppf)Cl_2_ (0.1 eq), and K_2_CO_3_ (3.0 eq) were combined in a microwave vessel equipped with a teflon stirbar. The system was flushed with argon. A degassed mixture of 3:1 acetone:water (3 mL) was added, and the reaction was heated in a microwave to 100 °C for 30 min. The product was purified via silica gel chromatography in ethyl acetate/hexanes.

#### 4.1.2. General Procedure B for HCl Boc Deprotection, Peptide Coupling, and TFA Boc Deprotection

The appropriate Boc-protected amine intermediate was dissolved in 1,4-dioxane (2–5 mL) and excess concentrated HCl (100–500 µL) was added. The reaction mixture stirred at room temperature for 1–3.5 h. The solvent was removed under vacuum to yield the deprotected amine. The amine intermediate (1.0 eq), diBoc-DMT (1.05 eq), PyBOP (1.0 eq), and 6Cl-HOBt (1.0 eq) were combined, and the reaction flask was flushed with argon. Dry DMF (3–12 mL) and DIEA (10 eq) were added. The reaction mixture stirred at room temperature for 6–24 h. The solvent was removed under vacuum, and the coupled product was purified via silica gel chromatography in ethyl acetate/hexanes. The Boc-protected compound was dissolved in DCM (2–2.5 mL). An equal volume of TFA was added, and the reaction mixture stirred at room temperature for 1–1.5 h. The solvent was removed under vacuum, and the product was purified by semi-preparative HPLC and lyophilized.

#### 4.1.3. General Procedure C for Microwave Suzuki Coupling of Boronic Ester **6** and Pendant Benzyl Bromide

The appropriate benzyl bromide (1.5–2.0 eq), intermediate **6** (1.0 eq), Pd(dppf)Cl_2_ (0.1 eq), and K_2_CO_3_ (3.0 eq) were combined in a microwave vessel equipped with a teflon stirbar. The system was flushed with argon. A degassed mixture of 3:1 acetone:water (2–3 mL) was added, and the reaction was heated in a microwave to 100 °C for 30 min. The product was purified via silica gel chromatography in ethyl acetate/hexanes.

#### 4.1.4. General Procedure D for Microwave Suzuki Coupling of Boronic Ester **6** and Pendant Benzyl Bromide

The appropriate benzyl bromide (1.0 eq), intermediate **6** (1.5 eq), Pd(dppf)Cl_2_ (0.1 eq), and K_2_CO_3_ (3.0 eq) were combined in a microwave vessel equipped with a teflon stirbar. The system was flushed with argon. A degassed mixture of 3:1 acetone:water (2–3 mL) was added, and the reaction was heated in a microwave to 100 °C for 30 min. The product was purified via silica gel chromatography in ethyl acetate/hexanes.

#### 4.1.5. General Procedure E for S_N_2 reaction of Benzyl Bromide **3** and Pendant Amine

Benzyl bromide **3** (1.0 eq), the appropriate nucleophile (1.2 eq), and K_2_CO_3_ (1.2 eq) were dissolved in dry DMF (3 mL) under an inert atmosphere. The reaction stirred at room temperature overnight. The reaction mixture was partitioned between 2 M NaOH and ethyl acetate. The aqueous layer was extracted with additional ethyl acetate. Combined organic layers were dried over MgSO_4,_ filtered, and concentrated under vacuum to obtain the product.

#### 4.1.6. General Procedure F for TFA Boc Deprotection, Peptide Coupling, and TFA Boc Deprotection

The appropriate Boc-protected amine intermediate was dissolved in DCM (1–3 mL). An equal volume of TFA was added, and the reaction mixture stirred at room temperature for 1–1.5 h. The solvent was removed under vacuum to yield the deprotected amine. The amine intermediate (1.0 eq), diBoc-DMT (1.05 eq), and PyBOP (1.0 eq) were combined, and the reaction flask was flushed with argon. Dry DMF (3–12 mL) and DIEA (10 eq) were added. The reaction mixture stirred at room temperature for 6–24 h. The solvent was removed under vacuum, and the coupled product was purified via silica gel chromatography in ethyl acetate/hexanes. The Boc-protected compound was dissolved in DCM (2–2.5 mL). An equal volume of TFA was added, and the reaction mixture stirred at room temperature for 1–1.5 h. The solvent was removed under vacuum, and the product was purified by semi-preparative HPLC and lyophilized.

*Tert-butyl 7-(hydroxymethyl)-3,4-dihydroisoquinoline-2(1H)-carboxylate* (**2**). To a solution of compound **1** in dry THF (15 mL), a 2.0 M solution of borane dimethyl sulfide in THF (2.7 mL, 5.41 mmol, 3.0 eq) was added dropwise over 15 min under inert atmosphere. The reaction mixture stirred at room temperature overnight. The reaction was quenched by the addition of methanol (20 mL). The solvent was removed under vacuum. The crude product was dissolved in ethyl acetate and washed with saturated aqueous NaHCO_3_ and brine. The combined aqueous layers were extracted with ethyl acetate. The combined organic layers were dried over MgSO_4_, filtered, and concentrated under vacuum to yield the product as a colorless oil (475 mg, 100%). ^1^H-NMR (CDCl_3_, 500 MHz) δ 7.16 (d, *J* = 7.9 Hz, 1H), 7.13 (d, *J* = 5.8 Hz, 1H), 7.12 (s, 1H), 4.65 (s, 2H), 4.57 (s, 2H), 3.63 (t, *J* = 5.9 Hz, 2H)), 2.82 (t, *J* = 5.8 Hz, 2H), 1.49 (s, 9H).

*Tert-butyl 7-(bromomethyl)-3,4-dihydroisoquinoline-2(1H)-carboxylate* (**3**). To a solution of compound **2** (950 mg, 3.61 mmol, 1.0 eq) in DCM (40 mL), CBr_4_ (1.32 g, 3.97 mmol, 1.1 eq) and a solution of PPh_3_ (1.14 g, 4.33 mmol, 1.2 eq) in DCM (5 mL) were added. The reaction stirred at room temperature for 2 h. The product was purified via silica gel chromatography in ethyl acetate/hexanes to yield a white solid (1.08 g, 92%). ^1^H-NMR (CDCl_3_, 500 MHz) δ 7.19 (d, *J* = 8.3 Hz, 1H), 7.14 (s, 1H), 7.11 (d, *J* = 7.8 Hz, 1H), 4.56 (s, 2H), 4.47 (s, 2H), 3.64 (t, *J* = 6.0 Hz, 2H), 2.82 (t, *J* = 5.9 Hz, 2H), 1.49 (s, 9H).

*Tert-butyl 7-bromo-3,4-dihydroisoquinoline-2(1H)-carboxylate* (**5**). 7-bromo-1,2,3,4-tetrahydroisoquinoline **4** (75 µL, 0.50 mmol, 1.0 eq) and di-tert-butyl dicarbonate (120 mg, 0.55 mmol, 1.1 eq) were combined in a microwave vessel equipped with a teflon stirbar. The system was flushed with argon, and the reaction was heated in a microwave to 100 °C for 15 min. The reaction mixture was diluted with DCM and washed with saturated aqueous NaHCO_3_ and brine. The organic layer was dried over MgSO_4,_ filtered, and concentrated under vacuum to obtain the product as a pale orange oil (145 mg, 99%). ^1^H-NMR (CDCl_3_, 500 MHz) δ 7.25 (m, 2H), 6.98 (d, *J* = 8.0 Hz, 1H), 4.52 (s, 2H), 3.61 (t, *J* = 5.7 Hz, 2H), 2.75 (t, *J* = 6.0 Hz, 2H), 1.48 (s, 9H).

*Tert-butyl 7-(4,4,5,5-tetramethyl-1,3,2-dioxaborolan-2-yl)-3,4-dihydroisoquinoline-2(1H)-carboxylate* (**6**). Intermediate **5** (945 mg, 3.03 mmol, 1.0 eq), bis(pinacolato)diboron (1.54 g, 6.06 mmol, 2.0 eq), Pd(dppf)Cl_2_ (222 mg, 0.303 mmol, 0.1 eq), and potassium acetate (892 mg, 9.09 mmol, 3.0 eq) were combined in DMSO (20 mL), and the system was flushed with argon. The reaction was heated to 90 °C overnight. The reaction mixture was concentrated under vacuum to remove most DMSO. The remaining mixture was diluted with water and extracted with three portions of DCM. The combined organic layers were washed with water and brine, dried over MgSO_4_, filtered, and concentrated under vacuum. The product was purified via silica gel chromatography in ethyl acetate/hexanes to yield a pale yellow oil (1.05 g, 96%). ^1^H-NMR (CDCl_3_, 500 MHz) δ 7.59 (d, *J* = 5.7 Hz, 1H), 7.14 (d, *J* = 5.5 Hz, 1H), 4.58 (s, 2H), 3.63 (br s, 2H), 2.84 (br s, 2H), 1.48 (s, 9H), 1.34 (s, 12H).

*Tert-butyl 7-(2-methoxybenzyl)-3,4-dihydroisoquinoline-2(1H)-carboxylate* (**7a**). Compound **7a** was synthesized following General Procedure A from compound **3** (60 mg, 0.18 mmol, 1.0 eq), (2-methoxyphenyl)boronic acid (42 mg, 0.28 mmol, 1.5 eq), Pd(dppf)Cl_2_ (13 mg, 0.02 mmol, 0.1 eq), and K_2_CO_3_ (76 mg, 0.55 mmol, 3.0 eq) to yield the product as a colorless oil (37 mg, 57%). ^1^H-NMR (CDCl_3_, 500 MHz) δ 7.21 (t, *J* = 7.6 Hz, 1H), 7.07 (d, *J* = 7.2 Hz, 1H), 7.03 (s, 2H), 6.95 (s, 1H), 6.88 (t, *J* = 8.2 Hz, 2H), 4.52 (s, 2H), 3.93 (s, 2H), 3.83 (s, 3H), 3.63 (s, 2H), 2.79 (t, *J* = 5.8 Hz, 2H), 1.49 (s, 9H).

*(S)-2-amino-3-(4-hydroxy-2,6-dimethylphenyl)-1-(7-(2-methoxybenzyl)-3,4-dihydroisoquinolin-2(1H)-yl)propan-1-one* (**8a**). Following General Procedure B, intermediate **7a** (37 mg, 0.10 mmol) was deprotected to yield the amine intermediate as a colorless oil. This intermediate was coupled to diBoc-DMT (45 mg, 0.10 mmol, 1.05 eq) in the presence of PyBOP (54 mg, 0.10 mmol, 1.0 eq), and DIEA (142 µL, 1.04 mmol, 10 eq) to yield the product as a brown oil. No 6Cl-HOBt was used. TFA deprotection yielded the product as a white solid. ^1^H-NMR (CD_3_OD, 500 MHz, rotamers) δ 7.17 (t, *J* = 7.9 Hz, 2H), 7.05 (t, *J* = 7.4 Hz, 2H), 6.97 (d, *J* = 7.7 Hz, 1H), 6.94–6.90 (m, 4H), 6.89–6.82 (m, 4H), 6.50 (d, *J* = 3.9 Hz, 1H), 6.39 (s, 2H), 6.33 (s, 2H), 4.59–4.45 (m, 4H), 4.15 (d, *J* = 15.7 Hz, 1H), 3.86 (d, *J* = 3.7 Hz, 2H), 3.85 (d, *J* = 2.8 Hz, 2H), 3.78 (d, *J* = 1.6 Hz, 3H), 3.78 (d, *J* = 1.2 Hz, 3H), 3.71–3.65 (m, 1H), 3.65–3.58 (m, 1H), 3.33 (d, *J* = 15.7 Hz, 1H), 3.25–3.17 (m, 2H), 3.09 (d, *J* = 11.4 Hz, 1H), 2.72–2.65 (m, 1H), 2.65–2.58 (m, 1H), 2.55–2.45 (m, 1H), 2.24 (s, 6H), 2.18 (s, 6H), 1.98–1.87 (m, 1H). HPLC retention time: 39.1 min. HREIMS *m*/*z* 445.2494 (calcd. for C28H32N2O3, 445.2486).

*Tert-butyl 7-(2-hydroxybenzyl)-3,4-dihydroisoquinoline-2(1H)-carboxylate* (**7b**). Compound **7b** was synthesized following General Procedure A from compound **3** (60 mg, 0.18 mmol, 1.0 eq), (2-hydroxyphenyl)boronic acid (38 mg, 0.28 mmol, 1.5 eq), Pd(dppf)Cl_2_ (13 mg, 0.02 mmol, 0.1 eq), and K_2_CO_3_ (76 mg, 0.55 mmol, 3.0 eq) to yield the product as a yellow oil (33 mg, 53%). ^1^H-NMR (CDCl_3_, 500 MHz) δ 7.16–7.10 (m, 2H), 7.08–7.01 (m, 2H), 6.96 (s, 1H), 6.89 (t, *J* = 7.5 Hz, 1H), 6.79 (d, *J* = 7.9 Hz, 1H), 4.51 (s, 2H), 3.95 (s, 2H), 3.62 (s, 2H), 2.78 (t, *J* = 5.9 Hz, 2H), 1.48 (s, 9H).

*(S)-2-amino-3-(4-hydroxy-2,6-dimethylphenyl)-1-(7-(2-hydroxybenzyl)-3,4-dihydroisoquinolin-2(1H)-yl)propan-1-one* (**8b**). Following General Procedure B, intermediate **7b** (33 mg, 0.10 mmol) was deprotected to yield the amine intermediate as an off-white solid. This intermediate was coupled to diBoc-DMT (42 mg, 0.10 mmol, 1.05 eq) in the presence of PyBOP (50 mg, 0.10 mmol, 1.0 eq), and DIEA (132 µL, 0.97 mmol, 10 eq) to yield the product as a brown oil. No 6Cl-HOBt was used. TFA deprotection yielded the product as a white solid. ^1^H-NMR (CD_3_OD, 500 MHz, rotamers) δ 7.01 (ddt, *J* = 10.9, 5.1, 1.8 Hz, 4H), 6.97 (d, *J* = 7.6 Hz, 3H), 6.92 (d, *J* = 7.9 Hz, 1H), 6.88 (d, *J* = 7.9 Hz, 1H), 6.78–6.70 (m, 4H), 6.55 (s, 1H), 6.40 (s, 2H), 6.33 (s, 2H), 4.61 (d, *J* = 16.9 Hz, 1H), 4.58–4.49 (m, 2H), 4.47 (d, *J* = 16.5 Hz, 1H), 4.16 (d, *J* = 15.6 Hz, 1H), 3.87 (s, 2H), 3.86 (s, 2H), 3.75–3.68 (m, 1H), 3.66–3.59 (m, 1H), 3.34 (d, *J* = 15.8 Hz, 1H), 3.26–3.17 (m, 3H), 3.10–3.05 (m, 2H), 2.72–2.65 (m, 2H), 2.65–2.59 (m, 2H), 2.55–2.45 (m, 1H), 2.24 (s, 6H), 2.19 (s, 6H), 2.03–1.95 (m, 2H). HPLC retention time: 32.8 min. HREIMS *m*/*z* 431.2337 (calcd. for C27H30N2O3, 431.2329).

*tert-Butyl 7-(2-(trifluoromethyl)benzyl)-3,4-dihydroisoquinoline-2(1H)-carboxylate* (**7c**). Compound **7c** was synthesized following General Procedure C from intermediate **6** (100 mg, 0.278 mmol, 1.0 eq), 1-(bromomethyl)-2-(trifluoromethyl)benzene (133 mg, 0.556 mmol, 2.0 eq), Pd(dppf)Cl_2_ (20 mg, 0.028 mmol, 0.1 eq), and K_2_CO_3_ (115 mg, 0.834 mmol, 3.0 eq) to yield a colorless oil (27 mg 25%). ^1^H-NMR (CDCl_3_, 400 MHz) δ 7.67 (d, *J* = 7.9 Hz, 1H), 7.43 (t, *J* = 7.5 Hz, 1H), 7.31 (t, *J* = 7.7 Hz, 1H), 7.18 (d, *J* = 7.9 Hz, 1H), 7.06 (d, *J* = 7.8 Hz, 1H), 6.95 (d, *J* = 7.0 Hz, 1H), 6.89 (s, 1H), 4.52 (s, 2H), 4.14 (s, 2H), 3.63 (t, *J* = 5.4 Hz, 2H), 2.80 (t, *J* = 5.9 Hz, 2H), 1.48 (s, 9H).

*(S)-2-amino-3-(4-hydroxy-2,6-dimethylphenyl)-1-(7-(2-(trifluoromethyl)benzyl)-3,4-dihydroisoquinolin-2(1H)-yl)propan-1-one* (**8c**). Following General Procedure B, intermediate **7c** (27 mg, 0.069 mmol, 1.0 eq) was deprotected to yield the amine intermediate as a white solid. This intermediate was coupled to diBoc-DMT (30 mg, 0.074 mmol, 1.05 eq) in the presence of PyBOP (36 mg, 0.070 mmol, 1.0 eq) and DIEA (98 μL, 0.700 mmol, 10 eq) to yield the product as a brown oil. No 6Cl-HOBt was used. TFA deprotection yielded the product as an off-white solid (24 mg, 57%, 3 steps). ^1^H-NMR (CD_3_OD, 500 MHz, rotamers) δ 7.70–7.66 (m, 2H), 7.54–7.48 (m, 2H), 7.40–7.35 (m, 2H), 7.28–7.22 (m, 2H), 6.98 (d, *J* = 7.8 Hz, 1H), 6.95–6.91 (m, 2H), 6.89 (s, 1H), 6.86 (d, 1H), 6.47 (s, 1H), 6.41 (s, 2H), 6.31 (s, 2H), 4.61 (d, *J* = 17.0 Hz, 1H), 4.58–4.51 (m, 2H), 4.49 (d, *J* = 17.0 Hz, 1H), 4.18 (d, *J* = 15.8 Hz, 1H), 4.12 (s, 2H), 4.11 (s, 2H), 3.86–3.80 (m, 1H), 3.59–3.52 (m, 1H), 3.39 (d, *J* = 15.8 Hz, 1H), 3.26–3.18 (m, 3H), 3.11–3.06 (m, 2H), 2.76–2.69 (m, 2H), 2.68–2.63 (m, 1H), 2.56–2.49 (m, 1H), 2.24 (s, 6H), 2.20 (s, 6H), 2.01–1.92 (m, 1H). ^19^F-NMR (CD_3_OD, 470 MHz, rotamers) δ −60.76, −77.10. HPLC retention time: 42.2 min. HREIMS *m*/*z* 483.2262 (calcd. for C28H29F3N2O2, 483.2254).

*tert-Butyl 7-(2-cyanobenzyl)-3,4-dihydroisoquinoline-2(1H)-carboxylate* (**7d**). Compound **7d** was synthesized following General Procedure C from intermediate **6** (75 mg, 0.209 mmol, 1.0 eq), 3-(bromomethyl)phenol (78 mg, 0.418 mmol, 2.0 eq), Pd(dppf)Cl_2_ (15 mg, 0.021 mmol, 0.1 eq), and K_2_CO_3_ (87 mg, 0.627 mmol, 3.0 eq) to yield the product as a colorless oil (26 mg, 37%). ^1^H-NMR (CDCl_3_, 500 MHz) δ 7.15 (t, *J* = 7.8 Hz, 1H), 7.04 (d, *J* = 7.8 Hz, 1H), 6.99 (d, *J* = 7.3 Hz, 1H), 6.92 (s, 1H), 6.79–6.74 (m, 1H), 6.69 (d, *J* = 8.1 Hz, 1H), 6.65 (s, 1H), 4.52 (s, 2H), 3.87 (s, 2H), 3.62 (t, *J* = 5.9 Hz, 2H), 2.78 (t, *J* = 5.9 Hz, 2H), 1.49 (s, 9H).

*(S)-2-((2-(2-amino-3-(4-hydroxy-2,6-dimethylphenyl)propanoyl)-1,2,3,4-tetrahydroisoquinolin-7-yl)methyl)benzonitrile* (**8d**). Following General Procedure B, intermediate **7d** (20 mg, 0.057 mmol, 1.0 eq) was deprotected to yield the amine intermediate as a colorless oil. This intermediate was coupled to diBoc-DMT (24 mg, 0.059 mmol, 1.05 eq) in the presence of PyBOP (29 mg, 0.056 mmol, 1.0 eq) and DIEA (80 μL, 0.560 mmol, 10 eq) to yield the product as a brown oil. No 6Cl-HOBt was used. TFA deprotection yielded the product as a white solid (3 mg, 10%, 3 steps). ^1^H-NMR (CD_3_OD, 500 MHz, rotamers) δ 7.70 (s, 1H), 7.68 (s, 1H), 7.62–7.57 (m, 2H), 7.45–7.35 (m, 4H), 7.03 (d, *J* = 8.1 Hz, 1H), 7.00 (s, 1H), 6.99–6.97 (m, 2H), 6.95 (d, *J* = 7.8 Hz, 1H), 6.56 (s, 1H), 6.39 (s, 2H), 6.29 (s, 2H), 4.62 (d, *J* = 16.9 Hz, 1H), 4.57–4.49 (m, 3H), 4.18 (d, *J* = 15.9 Hz, 1H), 4.15 (s, 2H), 4.13 (d, *J* = 3.1 Hz, 2H), 3.58–3.51 (m, 1H), 3.43 (d, *J* = 16.2 Hz, 0H), 3.26–3.18 (m, 4H), 3.06 (dd, *J* = 13.8, 4.2 Hz, 2H), 2.74–2.69 (m, 2H), 2.69–2.62 (m, 1H), 2.56–2.49 (m, 1H), 2.24 (s, 6H), 2.19 (s, 6H), 2.01–1.95 (m, 1H). HPLC retention time: 34.4 min. HREIMS *m*/*z* 440.2336 (calcd. for C28H29N3O2, 440.2333).

*Tert-butyl 7-(3-methoxybenzyl)-3,4-dihydroisoquinoline-2(1H)-carboxylate* (**7e**). Compound **7e** was synthesized following General Procedure D from 3-methoxybenzyl bromide (42 μL, 0.30 mmol, 1.0 eq), intermediate **6** (161 mg, 0.45 mmol, 1.5 eq), Pd(dppf)Cl_2_ (22 mg, 0.03 mmol, 0.1 eq), and K_2_CO_3_ (124 mg, 0.90 mmol, 3.0 eq) to yield the product as a colorless oil (42 mg, 40%). ^1^H-NMR (CDCl_3_, 500 MHz) δ 7.22 (t, *J* = 7.8 Hz, 1H), 7.06 (d, *J* = 7.8 Hz, 1H), 7.01 (d, *J* = 7.9 Hz, 1H), 6.94 (s, 1H), 6.79 (d, *J* = 7.5 Hz, 1H), 6.77–6.73 (m, 2H), 4.53 (s, 2H), 3.91 (s, 2H), 3.79 (s, 3H), 3.63 (br s, 2H), 2.80 (t, *J* = 5.9 Hz, 2H), 1.50 (s, 9H).

(*S)-2-amino-3-(4-hydroxy-2,6-dimethylphenyl)-1-(7-(3-methoxybenzyl)-3,4-dihydroisoquinolin-2(1H)-yl)propan-1-one* (**8e**). Following General Procedure B, intermediate **7e** (42 mg, 0.119 mmol, 1.0 eq) was deprotected to yield the amine intermediate as a white solid. This intermediate was coupled to diBoc-DMT (50 mg, 0.123 mmol, 1.05 eq) in the presence of PyBOP (61 mg, 0.117 mmol, 1.0 eq), 6Cl-HOBt (20 mg, 0.117 mmol, 1.0 eq), and DIEA (164 µL, 1.17 mmol, 10 eq). Silica gel chromatography yielded the coupled product as a colorless oil (66 mg, 88%). TFA deprotection yielded the product as a white solid. ^1^H-NMR (CD_3_OD, 500 MHz, rotamers) δ 7.16 (td, *J* = 7.8, 3.5 Hz, 2H), 6.99 (dd, *J* = 7.7, 1.7 Hz, 1H), 6.97–6.93 (m, 3H), 6.91 (d, *J* = 7.8 Hz, 1H), 6.76–6.70 (m, 6H), 6.49 (s, 1H), 6.40 (s, 2H), 6.32 (s, 2H), 4.61 (d, *J* = 16.9 Hz, 1H), 4.58–4.53 (m, 2H), 4.50 (d, *J* = 16.9 Hz, 1H), 4.17 (d, *J* = 15.7 Hz, 1H), 3.87 (s, 2H), 3.86 (s, 2H), 3.78–3.76 (m, 1H), 3.75 (s, 3H), 3.74 (s, 3H), 3.66–3.53 (m, 1H), 3.37 (d, *J* = 15.8 Hz, 1H), 3.26–3.17 (m, 3H), 3.11–3.05 (m, 2H), 2.70 (q, *J* = 7.3, 6.7 Hz, 2H), 2.67–2.62 (m, 1H), 2.52 (dt, *J* = 16.2, 6.2 Hz, 1H), 2.24 (s, 6H), 2.19 (s, 6H), 2.01–1.94 (m, 1H). HPLC retention time: 37.9 min. EIMS *m*/*z* 445.3 (calcd. for C28H32N2O3, 445.24).

*tert-Butyl 7-(3-hydroxybenzyl)-3,4-dihydroisoquinoline-2(1H)-carboxylate* (**7f**). Compound **7f** was synthesized following General Procedure C from intermediate **6** (75 mg, 0.209 mmol, 1.0 eq), 3-(bromomethyl)phenol (78 mg, 0.418 mmol, 2.0 eq), Pd(dppf)Cl_2_ (15 mg, 0.021 mmol, 0.1 eq), and K_2_CO_3_ (87 mg, 0.627 mmol, 3.0 eq) to yield the product as a colorless oil (26 mg, 37%). ^1^H-NMR (CDCl_3_, 500 MHz) δ 7.15 (t, *J* = 7.8 Hz, 1H), 7.04 (d, *J* = 7.8 Hz, 1H), 6.99 (d, *J* = 7.3 Hz, 1H), 6.92 (s, 1H), 6.79–6.74 (m, 1H), 6.69 (d, *J* = 8.1 Hz, 1H), 6.65 (s, 1H), 4.52 (s, 2H), 3.87 (s, 2H), 3.62 (t, *J* = 5.9 Hz, 2H), 2.78 (t, *J* = 5.9 Hz, 2H), 1.49 (s, 9H).

*(S)-2-amino-3-(4-hydroxy-2,6-dimethylphenyl)-1-(7-(3-hydroxybenzyl)-3,4-dihydroisoquinolin-2(1H)-yl)propan-1-one* (**8f**). Following General Procedure B, intermediate **7f** (26 mg, 0.077 mmol, 1.0 eq) was deprotected to yield the amine intermediate as a colorless oil. This intermediate was coupled to diBoc-DMT (33 mg, 0.080 mmol, 1.05 eq) in the presence of PyBOP (40 mg, 0.076 mmol, 1.0 eq) and DIEA (110 μL, 0.760 mmol, 10 eq) to yield the crude product as a brown oil. No 6Cl-HOBt was used. The crude product was purified via silica gel chromatography in ethyl acetate/hexanes. Subsequent TFA deprotection yielded the product as a white solid (8 mg, 40%, 3 steps). ^1^H-NMR (CD_3_OD, 500 MHz, rotamers) δ 7.09–7.03 (m, 2H), 6.99 (d, *J* = 8.5 Hz, 1H), 6.96–6.93 (m, 3H), 6.91 (d, *J* = 7.8 Hz, 1H), 6.65 (s, 1H), 6.64 (s, 1H), 6.61–6.57 (m, 3H), 6.49 (s, 1H), 6.40 (s, 2H), 6.32 (s, 2H), 4.62 (d, *J* = 16.9 Hz, 1H), 4.58–4.52 (m, 2H), 4.49 (d, *J* = 16.7 Hz, 1H), 4.17 (d, *J* = 15.7 Hz, 1H), 3.82 (s, 2H), 3.81 (s, 2H), 3.79–3.72 (m, 1H), 3.62–3.56 (m, 1H), 3.37 (d, *J* = 15.8 Hz, 1H), 3.26–3.17 (m, 3H), 3.07 (dd, *J* = 13.7, 4.2 Hz, 2H), 2.75–2.67 (m, 2H), 2.67–2.60 (m, 1H), 2.55–2.48 (m, 1H), 2.24 (s, 6H), 2.20 (s, 6H), 2.02–1.95 (m, 1H). HPLC retention time: 30.9 min. HREIMS *m*/*z* 431.2331 (calcd. for C27H30N2O3, 431.2329).

*Tert-butyl 7-(3-(trifluoromethyl)benzyl)-3,4-dihydroisoquinoline-2(1H)-carboxylate* (**7g**). Compound **7g** was synthesized following General Procedure D from 3-(trifluromethyl)benzyl bromide (33 μL, 0.21 mmol, 1.0 eq), intermediate **6** (114 mg, 0.32 mmol, 1.5 eq), Pd(dppf)Cl_2_ (15 mg, 0.02 mmol, 0.1 eq), and K_2_CO_3_ (88 mg, 0.64 mmol, 3.0 eq) to yield the product as a colorless oil (28 mg, 34%). EIMS *m*/*z* 414.2 (calcd. for C22H24F3NO2 + Na, 414.17).

*(S)-2-amino-3-(4-hydroxy-2,6-dimethylphenyl)-1-(7-(3-(trifluoromethyl)benzyl)-3,4-dihydroisoquinolin-2(1H)-yl)propan-1-one* (**8g**). Following General Procedure B, intermediate **7g** (28 mg, 0.072 mmol, 1.0 eq) was deprotected to yield the amine intermediate as a yellow oil. The crude product was rinsed with two 2 mL portions of diethyl ether to yield a white solid. This intermediate was coupled to diBoc-DMT (56 mg, 0.138 mmol, 1.92 eq) in the presence of PyBOP (68 mg, 0.131 mmol, 1.82 eq), 6Cl-HOBt (22 mg, 0.131 mmol, 1.82 eq), and DIEA (184 µL, 1.31 mmol, 18 eq). Silica gel chromatography yielded the coupled product as a colorless oil (64 mg, 72%). TFA deprotection yielded the product as a white solid. ^1^H-NMR (CD_3_OD, 500 MHz, rotamers) δ 7.50–7.43 (m, 8H), 7.02–6.97 (m, 3H), 6.96–6.93 (m, 2H), 6.48 (s, 1H), 6.39 (s, 2H), 6.30 (s, 2H), 4.62 (d, *J* = 16.9 Hz, 1H), 4.59–4.51 (m, 2H), 4.52 (d, *J* = 17.3 Hz, 1H), 4.18 (d, *J* = 15.8 Hz, 1H), 4.00 (s, 2H), 3.99 (s, 2H), 3.81 (dt, *J* = 12.9, 5.7 Hz, 1H), 3.59–3.52 (m, 1H), 3.40 (d, *J* = 15.8 Hz, 1H), 3.26–3.17 (m, 3H), 3.08 (dt, *J* = 13.8, 4.1 Hz, 2H), 2.71 (q, *J* = 5.4 Hz, 2H), 2.68–2.64 (m, 1H), 2.56–2.49 (m, 1H), 2.23 (s, 6H), 2.19 (s, 6H), 2.00–1.93 (m, 1H). ^19^F NMR (CD_3_OD, 470 MHz, rotamers) δ −64.03 (d, *J* = 28.1 Hz), −77.17. HPLC retention time: 43.3 min. EIMS *m*/*z* 483.3 (calcd. for C28H29F3N2O2, 483.22).

*Tert-butyl 7-(3-cyanobenzyl)-3,4-dihydroisoquinoline-2(1H)-carboxylate* (**7h**). Compound **7h** was synthesized following General Procedure D from 3-(bromomethyl)benzonitrile (40 mg, 0.20 mmol, 1.0 eq), intermediate **6** (109 mg, 0.30 mmol, 1.5 eq), Pd(dppf)Cl_2_ (15 mg, 0.02 mmol, 0.1 eq), and K_2_CO_3_ (84 mg, 0.61 mmol, 3.0 eq) to yield the product as a colorless oil (30 mg, 43%). ^1^H-NMR (CDCl_3_, 500 MHz) δ 7.50 (dt, *J* = 7.4, 1.5 Hz, 1H), 7.45 (s, 1H), 7.43 (t, *J* = 8.0 Hz, 1H), 7.39 (t, *J* = 7.6 Hz, 2H), 7.08 (d, *J* = 7.8 Hz, 1H), 6.96 (d, *J* = 7.7 Hz, 1H), 6.90 (s, 1H), 4.54 (s, 2H), 3.96 (s, 2H), 3.64 (t, *J* = 5.9 Hz, 2H), 2.81 (t, *J* = 5.9 Hz, 2H), 1.49 (s, 9H).

*(S)-2-amino-3-(4-hydroxy-2,6-dimethylphenyl)-1-(7-(3-isocyanobenzyl)-3,4-dihydroisoquinolin-2(1H)-yl)propan-1-one* (**8h**). Following General Procedure B, intermediate **7h** (30 mg, 0.086 mmol, 1.0 eq) was deprotected to yield the amine intermediate as an off-white solid (21 mg, 84%). The crude product was rinsed with three small portions of diethyl ether. This intermediate was coupled to diBoc-DMT (32 mg, 0.077 mmol, 1.05 eq) in the presence of PyBOP (39 mg, 0.074 mmol, 1.0 eq), 6Cl-HOBt (13 mg, 0.074 mmol, 1.0 eq), and DIEA (104 µL, 0.74 mmol, 10 eq). Silica gel chromatography yielded the coupled product as a white solid (27 mg, 57%). TFA deprotection yielded the product as a white solid. ^1^H-NMR (CD_3_OD, 500 MHz, rotamers) δ 7.57–7.51 (m, 6H), 7.48–7.43 (m, 2H), 7.03–6.99 (m, 3H), 6.97–6.94 (m, 2H), 6.48 (s, 1H), 6.39 (s, 2H), 6.29 (s, 2H), 4.63 (d, *J* = 17.0 Hz, 1H), 4.59–4.49 (m, 2H), 4.53 (d, *J* = 17.6 Hz, 2H), 4.18 (d, *J* = 15.8 Hz, 1H), 3.98 (s, 2H), 3.97 (s, 2H), 3.83 (dt, *J* = 12.3, 5.6 Hz, 1H), 3.59–3.51 (m, 1H), 3.41 (d, *J* = 15.7 Hz, 1H), 3.27–3.18 (m, 3H), 3.07 (dt, *J* = 13.8, 4.0 Hz, 2H), 2.74–2.70 (m, 2H), 2.69–2.63 (m, 1H), 2.53 (dt, *J* = 16.2, 5.9 Hz, 1H), 2.24 (s, 6H), 2.20 (s, 6H), 1.98 (dt, *J* = 16.0, 5.8 Hz, 1H). HPLC retention time: 35.2 min. EIMS *m*/*z* 440.2 (calcd. for C28H29N3O2, 440.23).

*Tert-butyl 7-(2,3-dimethylbenzyl)-3,4-dihydroisoquinoline-2(1H)-carboxylate* (**7i**). Compound **7i** was synthesized following General Procedure C from intermediate **6** (100 mg, 0.278 mmol, 1.0 eq), 1-(bromomethyl)-2,3-dimethylbenzene (83 mg, 0.417 mmol, 1.5 eq), Pd(dppf)Cl_2_ (20 mg, 0.028 mmol, 0.1 eq), and K_2_CO_3_ (115 mg, 0.834 mmol, 3.0 eq) to yield the product as a colorless oil (51 mg, 52%). ^1^H-NMR (CDCl_3_, 500 MHz) δ 7.09–7.06 (m, 2H), 7.04 (d, *J* = 7.6 Hz, 1H), 6.99 (d, *J* = 6.1 Hz, 1H), 6.93 (d, *J* = 7.9 Hz, 1H), 6.86 (s, 1H), 4.51 (s, 2H), 3.98 (s, 2H), 3.63 (t, *J* = 6.8 Hz, 2H), 2.79 (t, *J* = 6.0 Hz, 2H), 2.30 (s, 3H), 2.15 (s, 3H), 1.50 (s, 9H).

*(S)-2-amino-1-(7-(2,3-dimethylbenzyl)-3,4-dihydroisoquinolin-2(1H)-yl)-3-(4-hydroxy-2,6-dimethylphenyl)propan-1-one* (**8i**). Following General Procedure B, intermediate **7i** (51 mg, 0.145 mmol) was deprotected to yield the amine intermediate. This intermediate was coupled to diBoc-DMT (63 mg, 0.153 mmol, 1.05 eq) in the presence of PyBOP (76 mg, 0.146 mmol, 1.0 eq), and DIEA (199 µL, 1.46 mmol, 10 eq) to yield the product as a brown oil. No 6Cl-HOBt was used. TFA deprotection yielded the product as a white solid. ^1^H-NMR (CD_3_OD, 500 MHz, rotamers) δ 7.04–6.97 (m, 5H), 6.97–6.92 (m, 2H), 6.90–6.88 (m, 2H), 6.86–6.82 (m, 2H), 6.41 (s, 1H), 6.40 (s, 2H), 6.30 (s, 2H), 4.58 (d, *J* = 17.0 Hz, 1H), 4.62–4.48 (m, 2H), 4.46 (d, *J* = 16.9 Hz, 1H), 4.14 (d, *J* = 15.7 Hz, 1H), 3.95 (d, *J* = 2.8 Hz, 2H), 3.93 (s, 2H), 3.83–3.74 (m, 1H), 3.62–3.49 (m, 1H), 3.36 (d, *J* = 14.8 Hz, 1H), 3.26–3.17 (m, 3H), 3.08 (t, *J* = 4.9 Hz, 1H), 3.05 (t, *J* = 4.9 Hz, 1H), 2.72–2.61 (m, 3H), 2.54–2.47 (m, 1H), 2.25 (s, 3H), 2.25 (s, 3H), 2.23 (s, 6H), 2.19 (s, 6H), 2.09 (d, *J* = 2.2 Hz, 3H), 2.07 (d, *J* = 1.6 Hz, 3H), 2.00–1.93 (m, 1H). HPLC retention time: 43.2 min. HREIMS *m*/*z* 443.2699 (calcd. for C29H34N2O2, 443.2693).

*Tert-butyl 7-(pyridin-3-ylmethyl)-3,4-dihydroisoquinoline-2(1H)-carboxylate* (**7j**). Compound **7j** was synthesized following General Procedure C from intermediate **6** (104 mg, 0.289 mmol, 1.0 eq), 3-(bromomethyl)pyridine hydrobromide (110 mg, 0.433 mmol, 1.5 eq), Pd(dppf)Cl_2_ (21 mg, 0.029 mmol, 0.1 eq), and K_2_CO_3_ (120 mg, 0.867 mmol, 3.0 eq) to yield the product as a colorless oil (20 mg, 21%).^1^H-NMR (CDCl_3_, 500 MHz) δ 8.49 (s, 1H), 8.46 (d, *J* = 4.1 Hz, 1H), 7.46 (d, *J* = 7.7 Hz, 1H), 7.20 (dd, *J* = 7.8, 4.8 Hz, 1H), 7.07 (d, *J* = 7.9 Hz, 1H), 6.97 (d, *J* = 7.9 Hz, 1H), 6.91 (s, 1H), 4.52 (s, 2H), 3.93 (s, 2H), 3.62 (br s, 2H), 2.79 (t, *J* = 6.0 Hz, 2H), 1.48 (s, 9H).

*(S)-2-amino-3-(4-hydroxy-2,6-dimethylphenyl)-1-(7-(pyridin-3-ylmethyl)-3,4-dihydroisoquinolin-2(1H)-yl)propan-1-one* (**8j**). Following General Procedure B, intermediate **7j** (20 mg, 0.062 mmol, 1.0 eq) was deprotected to yield the amine intermediate as a colorless oil. The crude product was rinsed with several small portions of diethyl ether. This intermediate was coupled to diBoc-DMT (26 mg, 0.064 mmol, 1.05 eq) in the presence of PyBOP (32 mg, 0.061 mmol, 1.0 eq), 6Cl-HOBt (10 mg, 0.061 mmol, 1.0 eq), and DIEA (86 µL, 0.61 mmol, 10 eq). Silica gel chromatography yielded the coupled product (8 mg, 21%, 2 steps). TFA deprotection yielded the product as a white solid. ^1^H-NMR (CD_3_OD, 500 MHz, rotamers) δ 8.67–8.61 (m, 4H), 8.29 (d, *J* = 8.1 Hz, 1H), 8.26 (d, *J* = 8.0 Hz, 1H), 7.87 (dd, *J* = 8.1, 5.5 Hz, 1H), 7.83 (dd, *J* = 8.0, 5.5 Hz, 1H), 7.09–6.98 (m, 5H), 6.56 (s, 1H), 6.39 (s, 2H), 6.23 (s, 2H), 4.64 (d, *J* = 17.1 Hz, 1H), 4.59–4.52 (m, 2H), 4.53 (d, *J* = 16.6 Hz, 1H), 4.21 (d, *J* = 15.8 Hz, 1H), 4.14 (s, 2H), 4.12 (d, *J* = 3.2 Hz, 2H), 3.97 (dt, *J* = 13.2, 5.3 Hz, 1H), 3.48 (d, *J* = 16.1 Hz, 1H), 3.43 (dd, *J* = 13.0, 6.5 Hz, 1H), 3.26–3.19 (m, 3H), 3.08 (ddd, *J* = 13.7, 6.4, 4.1 Hz, 2H), 2.72 (t, *J* = 6.0 Hz, 2H), 2.69–2.62 (m, 1H), 2.57–2.49 (m, 1H), 2.23 (s, 6H), 2.20 (s, 6H), 1.99 (dt, *J* = 16.1, 6.1 Hz, 1H). HPLC retention time: 16.9 min. EIMS *m*/*z* 416.2 (calcd. for C26H29N3O2, 416.23).

*Tert-butyl 7-(pyridin-4-ylmethyl)-3,4-dihydroisoquinoline-2(1H)-carboxylate* (**7k**). Compound **7k** was synthesized following General Procedure C from intermediate **6** (112 mg, 0.312 mmol, 1.0 eq), 4-(bromomethyl)pyridine hydrobromide (118 mg, 0.468 mmol, 1.5 eq), Pd(dppf)Cl_2_ (23 mg, 0.031 mmol, 0.1 eq), and K_2_CO_3_ (129 mg, 0.936 mmol, 3.0 eq) to yield the product as a colorless oil (20 mg, 20%). ^1^H-NMR (CDCl_3_, 500 MHz) δ 8.49 (d, *J* = 5.4 Hz, 2H), 7.13–7.05 (m, 3H), 6.97 (d, *J* = 7.8 Hz, 1H), 6.91 (s, 1H), 4.53 (s, 2H), 3.91 (s, 2H), 3.63 (s, 2H), 2.80 (t, *J* = 5.0 Hz, 2H), 1.48 (s, 9H).

*(S)-2-amino-3-(4-hydroxy-2,6-dimethylphenyl)-1-(7-(pyridin-4-ylmethyl)-3,4-dihydroisoquinolin-2(1H)-yl)propan-1-one* (**8k**). Following General Procedure B, intermediate **7k** (20 mg, 0.062 mmol, 1.0 eq) was deprotected to yield the amine intermediate as a cloudy, yellow oil. The crude product was rinsed with several small portions of diethyl ether. This intermediate was coupled to diBoc-DMT (26 mg, 0.064 mmol, 1.05 eq) in the presence of PyBOP (32 mg, 0.061 mmol, 1.0 eq), 6Cl-HOBt (10 mg, 0.061 mmol, 1.0 eq), and DIEA (86 µL, 0.61 mmol, 10 eq). Silica gel chromatography yielded the coupled product (24 mg, 63%, 2 steps). TFA deprotection yielded the product as a white solid. ^1^H-NMR (CD_3_OD, 500 MHz, rotamers) δ 8.69–8.65 (m, 4H), 7.81–7.77 (m, 4H), 7.10–7.07 (m, 2H), 7.06 (d, *J* = 7.8 Hz, 1H), 7.01 (d, 2H), 6.58 (s, 1H), 6.40 (s, 2H), 6.25 (s, 2H), 4.65 (d, *J* = 17.1 Hz, 1H), 4.58–4.55 (m, 2H), 4.53 (d, *J* = 16.8 Hz, 1H), 4.25–4.22 (m, 3H), 4.20 (d, *J* = 3.0 Hz, 2H), 3.96 (dt, *J* = 12.9, 5.3 Hz, 1H), 3.48 (d, *J* = 15.0 Hz, 1H), 3.46–3.42 (m, 1H), 3.27–3.19 (m, 3H), 3.08 (ddd, *J* = 13.7, 6.2, 4.2 Hz, 2H), 2.74 (t, *J* = 6.1 Hz, 2H), 2.70–2.62 (m, 1H), 2.58–2.51 (m, 1H), 2.24 (s, 6H), 2.21 (s, 6H), 2.00 (dt, *J* = 16.2, 5.8 Hz, 1H). HPLC retention time: 17.0 min. EIMS *m*/*z* 416.3 (calcd. for C26H29N3O2, 416.23).

*Tert-butyl 7-(piperidin-1-ylmethyl)-3,4-dihydroisoquinoline-2(1H)-carboxylate* (**7l**). Compound **7l** was synthesized following General Procedure E from compound **3** (35 mg, 0.107 mmol, 1.0 eq), piperidine (13 μL, 0.129 mmol, 1.2 eq), and K_2_CO_3_ (18 mg, 0.129 mmol, 1.2 eq) to yield the product as an orange oil (27 mg, 77%). ^1^H-NMR (CDCl_3_, 500 MHz) δ 7.10 (d, *J* = 8.0 Hz, 1H), 7.09–7.03 (m, 2H), 4.56 (s, 2H), 3.63 (s, 2H), 3.42 (s, 2H), 2.80 (t, *J* = 5.7 Hz, 2H), 2.36 (s, 4H), 1.57 (p, *J* = 5.5 Hz, 4H), 1.48 (s, 9H), 1.47–1.39 (m, 2H).

*(S)-2-amino-3-(4-hydroxy-2,6-dimethylphenyl)-1-(7-(piperidin-1-ylmethyl)-3,4-dihydroisoquinolin-2(1H)-yl)propan-1-one* (**8l**). Following General Procedure B, intermediate **7l** (27 mg, 0.082 mmol, 1.0 eq) was deprotected to yield the amine intermediate as an orange oil. The crude product was rinsed with several small portions of diethyl ether. This intermediate was coupled to diBoc-DMT (35 mg, 0.087 mmol, 1.05 eq) in the presence of PyBOP (43 mg, 0.082 mmol, 1.0 eq), 6Cl-HOBt (14 mg, 0.082 mmol, 1.0 eq), and DIEA (115 µL, 0.82 mmol, 10 eq). TFA deprotection yielded the product as a white solid. ^1^H-NMR (CD_3_OD, 500 MHz, rotamers) δ 6.48–6.45 (m, 2H), 6.41 (d, *J* = 7.8 Hz, 1H), 6.35 (d, *J* = 7.8 Hz, 1H), 6.32 (d, *J* = 8.3 Hz, 1H), 5.97 (s, 1H), 5.59 (s, 2H), 5.41 (s, 2H), 3.89 (d, *J* = 17.3 Hz, 1H), 3.82–3.76 (m, 3H), 3.46 (d, *J* = 16.0 Hz, 1H), 3.43–3.32 (m, 4H), 3.24 (dt, *J* = 13.0, 5.2 Hz, 1H), 2.73 (d, *J* = 16.1 Hz, 2H), 2.67–2.56 (m, 5H), 2.46–2.37 (m, 3H), 2.32–2.25 (m, 2H), 2.10 (q, *J* = 10.9 Hz, 4H), 1.97 (t, *J* = 5.7 Hz, 2H), 1.93–1.85 (m, 1H), 1.79 (dt, *J* = 16.4, 6.5 Hz, 1H), 1.43 (s, 6H), 1.40 (s, 6H), 1.21 (dt, *J* = 16.4, 5.4 Hz, 1H), 1.13 (s, 2H), 1.10 (s, 3H), 1.01 (d, *J* = 14.4 Hz, 2H), 0.92 (q, *J* = 13.2 Hz, 4H), 0.74–0.66 (m, 2H). HPLC retention time: 16.7 min. EIMS *m*/*z* 422.3 (calcd. for C26H35N3O2, 422.27).

*Tert-butyl 7-(pyrrolidin-1-ylmethyl)-3,4-dihydroisoquinoline-2(1H)-carboxylate* (**7m**). Compound **7m** was synthesized following General Procedure E from compound **3** (29 mg, 0.089 mmol, 1.0 eq), pyrrolidine (9 μL, 0.107 mmol, 1.2 eq), and K_2_CO_3_ (15 mg, 0.107 mmol, 1.2 eq) to yield the product as a dark yellow-orange oil (28 mg, 100%). ^1^H-NMR (CDCl_3_, 500 MHz) δ 7.13–7.10 (m, 1H), 7.10–7.06 (m, 2H), 4.56 (s, 2H), 3.67–3.61 (m, 2H), 3.58 (s, 2H), 2.80 (d, *J* = 6.1 Hz, 2H), 2.55–2.47 (m, 4H), 1.79 (p, *J* = 3.0 Hz, 4H), 1.49 (s, 9H).

*(S)-2-amino-3-(4-hydroxy-2,6-dimethylphenyl)-1-(7-(pyrrolidin-1-ylmethyl)-3,4-dihydroisoquinolin-2(1H)-yl)propan-1-one* (**8m**). Following General Procedure B, intermediate **7m** (28 mg, 0.088 mmol, 1.0 eq) was deprotected to yield the amine intermediate. The crude product was rinsed with several small portions of diethyl ether. This intermediate was coupled to diBoc-DMT (37 mg, 0.091 mmol, 1.05 eq) in the presence of PyBOP (45 mg, 0.087 mmol, 1.0 eq), 6Cl-HOBt (15 mg, 0.087 mmol, 1.0 eq), and DIEA (122 µL, 0.87 mmol, 10 eq). TFA deprotection yielded the product as a white solid. ^1^H-NMR (CD_3_OD, 500 MHz, rotamers) δ 7.31–7.26 (m, 2H), 7.24 (d, *J* = 7.6 Hz, 2H), 7.17 (d, *J* = 7.9 Hz, 1H), 7.13 (d, *J* = 7.8 Hz, 1H), 6.79 (s, 1H), 6.41 (s, 2H), 6.23 (s, 2H), 4.70 (d, *J* = 17.2 Hz, 1H), 4.61 (d, *J* = 17.0 Hz, 1H), 4.62–4.51 (m, 2H), 4.31 (s, 2H), 4.31–4.22 (m, 3H), 4.03 (dt, *J* = 11.7, 5.2 Hz, 1H), 3.53 (d, *J* = 16.0 Hz, 1H), 3.51–3.41 (m, 5H), 3.29–3.25 (m, 1H), 3.26–3.21 (m, 2H), 3.20–3.13 (m, 4H), 3.12–3.06 (m, 2H), 2.79 (t, *J* = 6.0 Hz, 2H), 2.76–2.67 (m, 1H), 2.59 (dt, *J* = 16.4, 6.0 Hz, 1H), 2.24 (s, 6H), 2.22 (s, 6H), 2.19–2.16 (m, 4H), 2.05–1.97 (m, 5H). HPLC retention time: 15.3 min. HREIMS *m*/*z* 408.2649 (calcd. for C25H33N3O2, 408.2646).

*tert-butyl 7-(morpholinomethyl)-3,4-dihydroisoquinoline-2(1H)-carboxylate* (**7n**). Compound **7n** was synthesized following General Procedure E from compound **3** (31 mg, 0.095 mmol, 1.0 eq), morpholine (10 μL, 0.114 mmol, 1.2 eq), and K_2_CO_3_ (16 mg, 0.114 mmol, 1.2 eq) to yield the product as a pale yellow oil (32 mg, 100%). ^1^H-NMR (CDCl_3_, 500 MHz) δ 7.11 (d, *J* = 7.8 Hz, 1H), 7.08 (s, 2H), 4.56 (s, 2H), 3.70 (t, *J* = 4.7 Hz, 4H), 3.64 (t, *J* = 6.5 Hz, 2H), 3.45 (s, 2H), 2.81 (t, *J* = 5.9 Hz, 2H), 2.43 (t, *J* = 4.6 Hz, 4H), 1.49 (s, 9H).

*(S)-2-amino-3-(4-hydroxy-2,6-dimethylphenyl)-1-(7-(morpholinomethyl)-3,4-dihydroisoquinolin-2(1H)-yl)propan-1-one* (**8n**). Following General Procedure B, intermediate **7n** (28 mg, 0.088 mmol, 1.0 eq) was deprotected to yield the amine intermediate. The crude product was rinsed with several small portions of diethyl ether. This intermediate was coupled to diBoc-DMT (37 mg, 0.091 mmol, 1.05 eq) in the presence of PyBOP (45 mg, 0.087 mmol, 1.0 eq), 6Cl-HOBt (15 mg, 0.087 mmol, 1.0 eq), and DIEA (122 µL, 0.87 mmol, 10 eq). TFA deprotection yielded the product as a white solid. ^1^H-NMR (CD_3_OD, 500 MHz, rotamers) δ 7.30–7.28 (m, 2H), 7.24 (d, *J* = 7.8 Hz, 1H), 7.17 (d, *J* = 7.6 Hz, 1H), 7.14 (d, *J* = 7.8 Hz, 1H), 6.81 (s, 1H), 6.40 (s, 2H), 6.22 (s, 2H), 4.70 (d, *J* = 17.3 Hz, 1H), 4.63–4.55 (m, 3H), 4.31 (s, 2H), 4.29–4.21 (m, 3H), 4.13–4.05 (m, 1H), 4.08–3.99 (m, 4H), 3.74 (q, *J* = 12.7 Hz, 4H), 3.55 (d, *J* = 16.0 Hz, 1H), 3.42–3.32 (m, 5H), 3.28–3.25 (m, 1H), 3.25–3.20 (m, 2H), 3.21–3.12 (m, 4H), 3.13–3.06 (m, 2H), 2.79 (t, *J* = 6.1 Hz, 2H), 2.75–2.66 (m, 1H), 2.60 (dt, *J* = 16.5, 5.9 Hz, 1H), 2.24 (s, 6H), 2.22 (s, 6H), 2.03 (dt, *J* = 11.1, 5.7 Hz, 1H). HPLC retention time: 14.0 min. HREIMS *m*/*z* 424.2597 (calcd. for C25H33N3O3, 424.2595).

*Tert-butyl 7-(naphthalen-1-ylmethyl)-3,4-dihydroisoquinoline-2(1H)-carboxylate* (**7o**). Compound **7o** was synthesized following General Procedure C from intermediate **6** (100 mg, 0.278 mmol, 1.0 eq), 1-(bromomethyl)naphthalene (123 mg, 0.556 mmol, 2.0 eq), Pd(dppf)Cl_2_ (20 mg, 0.028 mmol, 0.1 eq), and K_2_CO_3_ (115 mg, 0.834 mmol, 3.0 eq) to yield the product as a yellow oil (41 mg, 39%). ^1^H-NMR (CDCl_3_, 500 MHz) δ 8.03–7.98 (m, 1H), 7.89–7.86 (m, 1H), 7.78 (d, *J* = 8.2 Hz, 1H), 7.49–7.45 (m, 2H), 7.43 (d, *J* = 7.7 Hz, 1H), 7.31 (d, *J* = 7.1 Hz, 1H), 7.03 (s, 2H), 6.95 (s, 1H), 4.50 (s, 2H), 4.41 (s, 2H), 3.63 (s, 2H), 2.80 (t, *J* = 5.9 Hz, 2H), 1.49 (s, 9H).

*(S)-2-amino-3-(4-hydroxy-2,6-dimethylphenyl)-1-(7-(naphthalen-1-ylmethyl)-3,4-dihydroisoquinolin-2(1H)-yl)propan-1-one* (**8o**). Following General Procedure B, intermediate **7o** (41 mg, 0.110 mmol, 1.0 eq) was deprotected to yield the amine intermediate. This intermediate was coupled to diBoc-DMT (47 mg, 0.115 mmol, 1.05 eq) in the presence of PyBOP (57 mg, 0.110 mmol, 1.0 eq), 6Cl-HOBt (57 mg, 0.330 mmol, 3.0 eq), and DIEA (154 µL, 1.1 mmol, 10 eq). Silica gel chromatography yielded the coupled product (57 mg, 78%, 2 steps). TFA deprotection yielded the product as a white solid. ^1^H-NMR (CD_3_OD, 500 MHz, rotamers) δ 7.98–7.94 (m, 2H), 7.86–7.83 (m, 2H), 7.76 (d, *J* = 8.2 Hz, 2H), 7.46–7.39 (m, 6H), 7.35 (d, *J* = 7.0 Hz, 1H), 7.32 (d, *J* = 6.9 Hz, 1H), 7.00 (d, *J* = 7.8 Hz, 1H), 6.97–6.94 (m, 2H), 6.92 (s, 1H), 6.88 (d, *J* = 7.8 Hz, 1H), 6.47 (s, 1H), 6.38 (s, 2H), 6.32 (s, 2H), 4.55 (d, *J* = 17.1 Hz, 1H), 4.52–4.47 (m, 2H), 4.44 (d, *J* = 16.9 Hz, 1H), 4.38 (s, 4H), 4.10 (d, *J* = 15.8 Hz, 1H), 3.79 (dt, *J* = 11.9, 5.6 Hz, 1H), 3.56–3.49 (m, 1H), 3.36 (d, *J* = 15.8 Hz, 1H), 3.24–3.15 (m, 3H), 3.05 (dt, *J* = 13.7, 5.1 Hz, 2H), 2.68 (t, *J* = 6.2 Hz, 2H), 2.66–2.60 (m, 1H), 2.49 (dt, *J* = 16.0, 6.2 Hz, 1H), 2.21 (s, 6H), 2.16 (s, 6H), 1.94 (dt, *J* = 16.1, 6.0 Hz, 1H). HPLC retention time: 43.3 min. EIMS *m*/*z* 465.2 (calcd. for C31H32N2O2, 465.25).

*Tert-butyl 7-(naphthalen-2-ylmethyl)-3,4-dihydroisoquinoline-2(1H)-carboxylate* (**7p**). Compound **7p** was synthesized following General Procedure C from intermediate **6** (100 mg, 0.278 mmol, 1.0 eq), 2-(bromomethyl)naphthalene (123 mg, 0.556 mmol, 2.0 eq), Pd(dppf)Cl_2_ (20 mg, 0.028 mmol, 0.1 eq), and K_2_CO_3_ (115 mg, 0.834 mmol, 3.0 eq) to yield the product (42 mg, 40%). ^1^H-NMR (CDCl_3_, 500 MHz) δ 7.79 (q, *J* = 8.1, 7.7 Hz, 3H), 7.65 (s, 1H), 7.45 (p, *J* = 7.1, 6.5 Hz, 2H), 7.32 (d, *J* = 8.5 Hz, 1H), 7.06 (s, 2H), 6.97 (s, 1H), 4.53 (s, 2H), 4.11 (s, 2H), 3.64 (s, 2H), 2.81 (t, *J* = 6.0 Hz, 2H), 1.49 (s, 9H).

*(S)-2-amino-3-(4-hydroxy-2,6-dimethylphenyl)-1-(7-(naphthalen-2-ylmethyl)-3,4-dihydroisoquinolin-2(1H)-yl)propan-1-one* (**8p**). Following General Procedure B, intermediate **7p** (42 mg, 0.112 mmol, 1.0 eq) was deprotected to yield the amine intermediate. Half of this intermediate (18 mg, 0.058 mmol, 1.0 eq) was coupled to diBoc-DMT (25 mg, 0.061 mmol, 1.05 eq) in the presence of PyBOP (30 mg, 0.058 mmol, 1.0 eq), 6Cl-HOBt (10 mg, 0.058 mmol, 1.0 eq), and DIEA (81 µL, 0.58 mmol, 10 eq). TFA deprotection yielded the product as a brown solid. ^1^H-NMR (CD_3_OD, 500 MHz, rotamers) δ 7.81–7.72 (m, 6H), 7.63 (d, *J* = 6.7 Hz, 2H), 7.46–7.38 (m, 4H), 7.29 (d, *J* = 8.3 Hz, 2H), 7.05 (d, *J* = 8.5 Hz, 1H), 7.02–6.96 (m, 3H), 6.93 (d, *J* = 7.8 Hz, 1H), 6.53 (s, 1H), 6.40 (s, 2H), 6.32 (s, 2H), 4.61 (d, *J* = 16.9 Hz, 1H), 4.57–4.48 (m, 2H), 4.50 (d, *J* = 16.8 Hz, 1H), 4.16 (d, *J* = 15.8 Hz, 1H), 4.07 (s, 2H), 4.06 (s, 2H), 3.77 (dt, *J* = 12.1, 5.7 Hz, 1H), 3.61–3.53 (m, 1H), 3.38 (d, *J* = 15.8 Hz, 1H), 3.24–3.17 (m, 3H), 3.06 (dt, *J* = 13.8, 5.2 Hz, 2H), 2.71 (q, *J* = 5.8 Hz, 2H), 2.67–2.61 (m, 1H), 2.52 (dt, *J* = 15.7, 6.3 Hz, 1H), 2.22 (s, 6H), 2.18 (s, 6H), 1.98 (dt, *J* = 16.2, 5.9 Hz, 1H). HPLC retention time: 43.5 min. EIMS *m*/*z* 465.3 (calcd. for C31H32N2O2, 465.25).

*Tert-butyl 7-(quinolin-8-ylmethyl)-3,4-dihydroisoquinoline-2(1H)-carboxylate* (**7q**). Compound **7q** was synthesized following General Procedure A from compound **3** (75 mg, 0.23 mmol, 1.0 eq), quinolin-8-ylboronic acid (60 mg, 0.345 mmol, 1.5 eq), Pd(dppf)Cl_2_ (17 mg, 0.023 mmol, 0.1 eq), and K_2_CO_3_ (95 mg, 0.69 mmol, 3.0 eq) to yield the product as a colorless oil (18 mg, 21%). ^1^H-NMR (CDCl_3_, 500 MHz) δ 8.97 (dd, *J* = 4.2, 1.8 Hz, 1H), 8.15 (dd, *J* = 8.3, 1.8 Hz, 1H), 7.74–7.65 (m, 1H), 7.46 (s, 1H), 7.44 (s, 1H), 7.41 (dd, *J* = 8.3, 4.2 Hz, 1H), 7.13 (dd, *J* = 7.6, 1.8 Hz, 1H), 7.07 (s, 1H), 7.04 (d, *J* = 7.9 Hz, 1H), 4.64 (s, 2H), 4.52 (s, 2H), 3.62 (s, 2H), 2.79 (t, *J* = 5.3 Hz, 2H), 1.48 (s, 9H).

*(S)-2-amino-3-(4-hydroxy-2,6-dimethylphenyl)-1-(7-(quinolin-8-ylmethyl)-3,4-dihydroisoquinolin-2(1H)-yl)propan-1-one* (**8q**). Following General Procedure F, intermediate **7q** (18 mg, 0.048 mmol) was deprotected to yield the amine intermediate. This intermediate was coupled to diBoc-DMT (21 mg, 0.05 mmol, 1.05 eq) in the presence of PyBOP (25 mg, 0.048 mmol, 1.0 eq), and DIEA (84 µL, 0.48 mmol, 10 eq) to yield the product. No 6Cl-HOBt was used. TFA deprotection yielded the product as a white solid. ^1^H-NMR (CD_3_OD, 500 MHz, rotamers) δ 9.03 (td, *J* = 4.9, 1.7 Hz, 2H), 8.79 (dd, *J* = 8.3, 1.7 Hz, 1H), 8.75 (dd, *J* = 8.3, 1.7 Hz, 1H), 8.04 (dd, *J* = 8.1, 1.6 Hz, 1H), 8.00 (dd, *J* = 8.1, 1.4 Hz, 1H), 7.81 (ddd, *J* = 13.0, 8.3, 4.9 Hz, 2H), 7.75–7.66 (m, 3H), 7.63 (dd, *J* = 7.3, 1.4 Hz, 1H), 7.04 (dd, *J* = 7.8, 1.8 Hz, 1H), 7.01 (d, *J* = 1.7 Hz, 1H), 6.96 (s, 2H), 6.94 (d, *J* = 7.9 Hz, 1H), 6.58 (s, 1H), 6.38 (s, 2H), 6.24 (s, 2H), 4.61 (d, *J* = 15.8 Hz, 1H), 4.58 (s, 2H), 4.56 (s, 2H), 4.56–4.52 (m, 2H), 4.47 (d, *J* = 17.0 Hz, 1H), 4.17 (d, *J* = 15.9 Hz, 1H), 3.84 (dt, *J* = 12.9, 5.5 Hz, 1H), 3.54–3.47 (m, 1H), 3.38 (d, *J* = 15.9 Hz, 1H), 3.25–3.16 (m, 3H), 3.11–3.05 (m, 2H), 2.69 (q, *J* = 5.5 Hz, 2H), 2.66–2.59 (m, 1H), 2.52 (dt, *J* = 16.2, 7.4, 4.8 Hz, 1H), 2.21 (s, 6H), 2.18 (s, 6H), 1.98 (ddd, *J* = 16.2, 6.4, 4.4 Hz, 1H). HPLC retention time: 25.0 min. EIMS *m*/*z* 466.3 (calcd. for C30H31N3O2, 466.24).

*Tert-butyl 7-(isoquinolin-8-ylmethyl)-3,4-dihydroisoquinoline-2(1H)-carboxylate* (**7r**). Compound **7r** was synthesized following General Procedure A from compound **3** (77 mg, 0.24 mmol, 1.0 eq), isoquinolin-8-ylboronic acid (49 mg, 0.283 mmol, 1.2 eq), Pd(dppf)Cl_2_ (18 mg, 0.024 mmol, 0.1 eq), and K_2_CO_3_ (98 mg, 0.71 mmol, 3.0 eq) to yield the product as a pale pink oil (55 mg, 62%). ^1^H-NMR (CDCl_3_, 500 MHz) δ 9.47 (s, 1H), 8.52 (d, *J* = 5.6 Hz, 1H), 7.73 (d, *J* = 8.3 Hz, 1H), 7.68–7.60 (m, 2H), 7.40 (d, *J* = 7.0 Hz, 1H), 7.02 (q, *J* = 8.0 Hz, 2H), 6.94 (s, 1H), 4.48 (s, 4H), 3.61 (s, 2H), 2.77 (t, *J* = 6.1 Hz, 2H), 1.47 (s, 9H).

*(S)-2-amino-3-(4-hydroxy-2,6-dimethylphenyl)-1-(7-(isoquinolin-8-ylmethyl)-3,4-dihydroisoquinolin-2(1H)-yl)propan-1-one* (**8r**). Following General Procedure F, intermediate **7r** (27 mg, 0.072 mmol) was deprotected to yield the amine intermediate. This intermediate was coupled to diBoc-DMT (31 mg, 0.076 mmol, 1.05 eq) in the presence of PyBOP (37 mg, 0.072 mmol, 1.0 eq), and DIEA (125 µL, 0.72 mmol, 10 eq) to yield the product. No 6Cl-HOBt was used. TFA deprotection yielded the product as a white solid. ^1^H-NMR (CD_3_OD, 500 MHz, rotamers) δ 9.73 (s, 1H), 9.72 (s, 1H), 8.54 (t, *J* = 5.0 Hz, 2H), 8.38 (d, *J* = 6.3 Hz, 1H), 8.35 (d, *J* = 6.2 Hz, 1H), 8.17–8.10 (m, 3H), 8.08 (q, *J* = 7.6 Hz, 1H), 7.82 (d, *J* = 6.6 Hz, 1H), 7.76 (d, *J* = 6.9 Hz, 1H), 7.07–7.02 (m, 2H), 7.00–6.94 (m, 3H), 6.53 (s, 1H), 6.36 (s, 2H), 6.17 (s, 2H), 4.61 (s, 2H), 4.60 (s, 2H), 4.57 (d, *J* = 14.3 Hz, 1H), 4.54 (dd, *J* = 11.9, 4.1 Hz, 2H), 4.48 (d, *J* = 17.0 Hz, 1H), 4.16 (d, *J* = 15.8 Hz, 1H), 3.97 (dt, *J* = 12.9, 5.3 Hz, 1H), 3.45 (d, *J* = 15.8 Hz, 1H), 3.38 (dt, *J* = 13.2, 6.7 Hz, 1H), 3.24–3.15 (m, 3H), 3.07 (ddd, *J* = 13.6, 11.5, 4.1 Hz, 2H), 2.69 (t, *J* = 6.0 Hz, 2H), 2.62 (ddd, *J* = 12.5, 7.5, 4.7 Hz, 1H), 2.52 (ddd, *J* = 16.2, 7.5, 4.8 Hz, 1H), 2.19 (s, 6H), 2.17 (s, 6H), 1.96 (ddd, *J* = 16.3, 6.9, 4.7 Hz, 1H). HPLC retention time: 21.4 min. EIMS *m*/*z* 466.3 (calcd. for C30H31N3O2, 466.24).

*Tert-butyl 7-(isoquinolin-5-ylmethyl)-3,4-dihydroisoquinoline-2(1H)-carboxylate* (**7s**). Compound **7s** was synthesized following General Procedure A from compound **3** (73 mg, 0.224 mmol, 1.0 eq), isoquinolin-5-ylboronic acid (46 mg, 0.269 mmol, 1.2 eq), Pd(dppf)Cl_2_ (16 mg, 0.022 mmol, 0.1 eq), and K_2_CO_3_ (93 mg, 0.672 mmol, 3.0 eq) to yield the product as a yellow oil (48 mg, 57%). ^1^H-NMR (CDCl_3_, 500 MHz) δ 9.26 (s, 1H), 8.50 (d, *J* = 6.0 Hz, 1H), 7.88 (d, *J* = 8.1 Hz, 1H), 7.76 (d, *J* = 6.0 Hz, 1H), 7.55 (t, *J* = 7.6 Hz, 1H), 7.50 (d, *J* = 7.1 Hz, 1H), 7.04 (d, *J* = 7.8 Hz, 1H), 6.97 (s, 1H), 6.91 (s, 1H), 4.49 (s, 2H), 4.36 (s, 2H), 3.62 (t, *J* = 5.9 Hz, 2H), 2.78 (t, *J* = 5.9 Hz, 2H), 1.47 (s, 9H).

*(S)-2-amino-3-(4-hydroxy-2,6-dimethylphenyl)-1-(7-(isoquinolin-5-ylmethyl)-3,4-dihydroisoquinolin-2(1H)-yl)propan-1-one* (**8s**). Following General Procedure F, intermediate **7s** (24 mg, 0.064 mmol) was deprotected to yield the amine intermediate. This intermediate was coupled to diBoc-DMT (28 mg, 0.067 mmol, 1.05 eq) in the presence of PyBOP (33 mg, 0.064 mmol, 1.0 eq), and DIEA (111 µL, 0.64 mmol, 10 eq) to yield the product. No 6Cl-HOBt was used. TFA deprotection yielded the product as a white solid. ^1^H-NMR (CD_3_OD, 500 MHz, rotamers) δ 9.67 (d, *J* = 7.1 Hz, 2H), 8.52 (d, *J* = 7.0 Hz, 2H), 8.44 (t, *J* = 7.6 Hz, 2H), 8.35 (t, *J* = 8.9 Hz, 2H), 8.04–7.91 (m, 4H), 7.04–6.92 (m, 5H), 6.51 (s, 1H), 6.37 (s, 2H), 6.23 (s, 2H), 4.58 (d, *J* = 17.4 Hz, 2H), 4.56–4.50 (m, 2H), 4.53 (s, 2H), 4.52 (s, 2H), 4.47 (d, *J* = 17.1 Hz, 1H), 4.15 (d, *J* = 15.8 Hz, 1H), 3.90 (dt, *J* = 12.9, 5.4 Hz, 1H), 3.48–3.39 (m, 1H), 3.41 (d, *J* = 15.8 Hz, 1H), 3.24–3.16 (m, 3H), 3.07 (ddd, *J* = 13.3, 8.5, 4.1 Hz, 2H), 2.69 (t, 2H), 2.62 (ddd, *J* = 12.6, 7.5, 4.7 Hz, 1H), 2.51 (ddd, *J* = 16.2, 7.3, 4.8 Hz, 1H), 2.21 (s, 6H), 2.17 (s, 6H), 1.96 (ddd, *J* = 16.2, 6.9, 4.9 Hz, 1H). HPLC retention time: 21.5 min. EIMS *m*/*z* 466.3 (calcd. for C30H31N3O2, 466.24).

*Tert-butyl 7-(quinolin-5-ylmethyl)-3,4-dihydroisoquinoline-2(1H)-carboxylate* (**7t**). Compound **7t** was synthesized following General Procedure A from compound **3** (50 mg, 0.153 mmol, 1.0 eq), quinolin-5-ylboronic acid (32 mg, 0.184 mmol, 1.2 eq), Pd(dppf)Cl_2_ (11 mg, 0.015 mmol, 0.1 eq), and K_2_CO_3_ (63 mg, 0.459 mmol, 3.0 eq) to yield the product as a yellow oil (36 mg, 63%). ^1^H-NMR (CDCl_3_, 500 MHz) δ 8.90 (dd, *J* = 4.2, 1.7 Hz, 1H), 8.30 (ddd, *J* = 8.6, 1.7, 0.9 Hz, 1H), 8.04 (d, *J* = 8.5 Hz, 1H), 7.66 (t, *J* = 7.8 Hz, 1H), 7.39–7.33 (m, 2H), 7.03 (d, *J* = 7.6 Hz, 1H), 6.99–6.85 (m, 2H), 4.48 (s, 2H), 4.39 (s, 2H), 3.60 (t, *J* = 6.1 Hz, 2H), 2.77 (t, *J* = 6.0 Hz, 2H), 1.47 (s, 9H).

*(S)-2-amino-3-(4-hydroxy-2,6-dimethylphenyl)-1-(7-(quinolin-5-ylmethyl)-3,4-dihydroisoquinolin-2(1H)-yl)propan-1-one* (**8t**). Following General Procedure F, intermediate **7t** (36 mg, 0.096 mmol) was deprotected to yield the amine intermediate. This intermediate was coupled to diBoc-DMT (41 mg, 0.100 mmol, 1.05 eq) in the presence of PyBOP (49 mg, 0.095 mmol, 1.0 eq), and DIEA (123 µL, 0.95 mmol, 10 eq) to yield the product. No 6Cl-HOBt was used. TFA deprotection yielded the product as a white solid. ^1^H-NMR (CD_3_OD, 500 MHz, rotamers) δ 9.15 (dd, *J* = 8.5, 5.5 Hz, 2H), 9.10 (td, *J* = 5.1, 1.5 Hz, 2H), 8.13 (t, *J* = 9.1 Hz, 2H), 8.05 (ddd, *J* = 18.3, 8.7, 7.1 Hz, 2H), 7.93 (ddd, *J* = 8.7, 7.4, 5.2 Hz, 2H), 7.78 (d, *J* = 7.1 Hz, 1H), 7.74 (d, *J* = 7.1 Hz, 1H), 7.04–6.92 (m, 5H), 6.50 (s, 1H), 6.36 (s, 2H), 6.23 (s, 2H), 4.59 (d, *J* = 16.4 Hz, 1H), 4.55 (s, 2H), 4.54 (s, 2H), 4.54–4.52 (m, 2H), 4.47 (d, *J* = 17.1 Hz, 1H), 4.15 (d, *J* = 15.8 Hz, 1H), 3.90 (dt, *J* = 12.9, 5.4 Hz, 1H), 3.43 (dt, *J* = 13.1, 6.6 Hz, 1H), 3.41 (d, *J* = 15.9 Hz, 1H), 3.24–3.16 (m, 3H), 3.07 (ddd, *J* = 13.3, 8.5, 4.1 Hz, 2H), 2.69 (t, *J* = 6.0 Hz, 2H), 2.63 (ddd, *J* = 12.5, 7.4, 4.7 Hz, 1H), 2.50 (ddd, *J* = 16.2, 7.4, 4.8 Hz, 1H), 2.20 (s, 6H), 2.17 (s, 6H), 1.94 (ddd, *J* = 16.1, 6.8, 4.6 Hz, 1H). HPLC retention time: 21.6 min. EIMS *m*/*z* 466.3 (calcd. for C30H31N3O2, 466.24).

*Tert-butyl 7-(quinolin-4-ylmethyl)-3,4-dihydroisoquinoline-2(1H)-carboxylate* (**7u**). Compound **7u** was synthesized following General Procedure A from compound **3** (50 mg, 0.153 mmol, 1.0 eq), quinolin-4-ylboronic acid (32 mg, 0.184 mmol, 1.2 eq), Pd(dppf)Cl_2_ (11 mg, 0.015 mmol, 0.1 eq), and K_2_CO_3_ (63 mg, 0.459 mmol, 3.0 eq) to yield the product as a colorless oil (32 mg, 56%). ^1^H-NMR (CDCl_3_, 500 MHz) δ 8.83 (d, *J* = 4.4 Hz, 1H), 8.13 (d, *J* = 8.4 Hz, 1H), 8.03 (dd, *J* = 8.5, 1.3 Hz, 1H), 7.70 (t, *J* = 7.7 Hz, 1H), 7.54 (t, *J* = 7.7 Hz, 1H), 7.15 (d, *J* = 4.2 Hz, 1H), 7.07 (d, *J* = 7.6 Hz, 1H), 7.03–6.88 (m, 2H), 4.50 (s, 2H), 4.40 (s, 2H), 3.63 (t, *J* = 5.4 Hz, 2H), 2.80 (t, *J* = 5.8 Hz, 2H), 1.47 (s, 9H).

*(S)-2-amino-3-(4-hydroxy-2,6-dimethylphenyl)-1-(7-(quinolin-4-ylmethyl)-3,4-dihydroisoquinolin-2(1H)-yl)propan-1-one* (**8u**). Following General Procedure F, intermediate **7u** (32 mg, 0.085 mmol) was deprotected to yield the amine intermediate. This intermediate was coupled to diBoc-DMT (37 mg, 0.089 mmol, 1.05 eq) in the presence of PyBOP (44 mg, 0.085 mmol, 1.0 eq), and DIEA (148 µL, 0.85 mmol, 10 eq) to yield the product. No 6Cl-HOBt was used. TFA deprotection yielded the product as a white solid. ^1^H-NMR (CD_3_OD, 500 MHz, rotamers) δ 9.07 (dd, *J* = 9.2, 5.6 Hz, 2H), 8.55 (t, *J* = 9.6 Hz, 2H), 8.26 (t, *J* = 8.8 Hz, 2H), 8.18–8.11 (m, 2H), 8.00–7.93 (m, 2H), 7.78 (d, *J* = 5.6 Hz, 2H), 7.12 (dd, *J* = 7.7, 1.8 Hz, 1H), 7.10 (s, 1H), 7.05 (s, 2H), 7.01 (d, *J* = 7.8 Hz, 1H), 6.60 (s, 1H), 6.37 (s, 2H), 6.20 (s, 2H), 4.74 (s, 2H), 4.71 (s, 2H), 4.61 (d, *J* = 17.2 Hz, 1H), 4.55 (dd, *J* = 12.0, 4.1 Hz, 2H), 4.51 (d, *J* = 17.1 Hz, 1H), 4.20 (d, *J* = 15.8 Hz, 1H), 3.99 (dt, *J* = 12.9, 5.3 Hz, 1H), 3.48 (d, *J* = 15.8 Hz, 1H), 3.40 (dt, *J* = 13.2, 6.7 Hz, 1H), 3.25–3.17 (m, 3H), 3.08 (ddd, *J* = 13.9, 9.9, 4.1 Hz, 2H), 2.73 (t, *J* = 6.1 Hz, 2H), 2.65 (ddd, *J* = 12.5, 7.4, 4.8 Hz, 1H), 2.54 (ddd, *J* = 16.4, 7.4, 4.9 Hz, 1H), 2.21 (s, 6H), 2.18 (s, 6H), 1.98 (ddd, *J* = 16.3, 7.0, 4.7 Hz, 1H). HPLC retention time: 21.2 min. EIMS *m*/*z* 466.3 (calcd. for C30H31N3O2, 466.24).

*Tert-butyl 7-(isoquinolin-4-ylmethyl)-3,4-dihydroisoquinoline-2(1H)-carboxylate* (**7v**). Compound **7v** was synthesized following General Procedure A from compound **3** (50 mg, 0.153 mmol, 1.0 eq), isoquinolin-4-ylboronic acid (32 mg, 0.184 mmol, 1.2 eq), Pd(dppf)Cl_2_ (11 mg, 0.015 mmol, 0.1 eq), and K_2_CO_3_ (63 mg, 0.459 mmol, 3.0 eq) to yield the crude product. Silica gel chromatography yielded a mixture of products. This mixture was used directly in the next step.

*(S)-2-amino-3-(4-hydroxy-2,6-dimethylphenyl)-1-(7-(isoquinolin-4-ylmethyl)-3,4-dihydroisoquinolin-2(1H)-yl)propan-1-one* (**8v**). Following General Procedure F, intermediate **7v** (19 mg, 0.051 mmol) was deprotected. The crude product was purified by semi-preparative HPLC to yield the product as a white solid (21 mg, 100%). EIMS calcd. for [C19H18N2 + H]^+^: 275.15, found: 275.2. The amine was coupled to diBoc-DMT (23 mg, 0.057 mmol, 1.05 eq) in the presence of PyBOP (28 mg, 0.054 mmol, 1.0 eq), and DIEA (94 µL, 0.54 mmol, 10 eq) to yield the product. No 6Cl-HOBt was used. TFA deprotection yielded the product as a white solid. ^1^H-NMR (CD_3_OD, 500 MHz, rotamers) δ 9.65 (d, *J* = 6.0 Hz, 2H), 8.50 (t, *J* = 8.3 Hz, 2H), 8.42–8.35 (m, 4H), 8.22–8.14 (m, 2H), 8.04–7.97 (m, 2H), 7.10 (dd, *J* = 7.8, 1.8 Hz, 1H), 7.07 (s, 1H), 7.02 (s, 2H), 6.97 (d, *J* = 7.9 Hz, 1H), 6.56 (s, 1H), 6.37 (s, 2H), 6.21 (s, 2H), 4.61 (d, *J* = 16.1 Hz, 1H), 4.57 (s, 2H), 4.56 (s, 2H), 4.55–4.52 (m, 2H), 4.49 (d, *J* = 17.2 Hz, 1H), 4.17 (d, *J* = 15.8 Hz, 1H), 3.95 (dt, *J* = 12.9, 5.3 Hz, 1H), 3.45 (d, *J* = 15.5 Hz, 1H), 3.43–3.38 (m, 1H), 3.25–3.17 (m, 3H), 3.07 (ddd, *J* = 13.7, 8.1, 4.1 Hz, 2H), 2.72 (t, *J* = 6.0 Hz, 2H), 2.65 (ddd, *J* = 12.7, 7.3, 4.8 Hz, 1H), 2.52 (ddd, *J* = 16.4, 7.1, 4.8 Hz, 1H), 2.21 (s, 6H), 2.17 (s, 6H), 1.95 (dt, *J* = 16.4, 6.2 Hz, 1H). HPLC retention time: 21.5 min. EIMS *m*/*z* 466.3 (calcd. for C30H31N3O2, 466.24).

*Tert-butyl 7-((3,4-dihydroisoquinolin-2(1H)-yl)methyl)-3,4-dihydroisoquinoline-2(1H)-carboxylate* (**7w**). Compound **7w** was synthesized following General Procedure E from compound **3** (50 mg, 0.153 mmol, 1.0 eq), 1,2,3,4-tetrahydroisoquinoline (23 μL, 0.184 mmol, 1.2 eq), and K_2_CO_3_ (25 mg, 0.184 mmol, 1.2 eq) to yield the product as a colorless oil (24 mg, 41%). ^1^H-NMR (CDCl_3_, 500 MHz) δ 7.19 (d, *J* = 7.5 Hz, 1H), 7.15 (s, 1H), 7.12–7.08 (m, 4H), 6.99 (d, *J* = 7.0 Hz, 1H), 4.57 (s, 2H), 3.66 (br s, 2H), 3.64 (s, 2H), 3.63 (s, 2H), 2.90 (t, *J* = 6.0 Hz, 2H), 2.83 (t, *J* = 5.9 Hz, 2H), 2.75 (t, *J* = 5.9 Hz, 2H), 1.49 (s, 9H).

*(S)-2-amino-1-(7-((3,4-dihydroisoquinolin-2(1H)-yl)methyl)-3,4-dihydroisoquinolin-2(1H)-yl)-3-(4-hydroxy-2,6-dimethylphenyl)propan-1-one* (**8w**). Following General Procedure F, intermediate **7w** (24 mg, 0.063 mmol, 1.0 eq) was deprotected to yield the amine intermediate. This intermediate was coupled to diBoc-DMT (27 mg, 0.067 mmol, 1.05 eq) in the presence of PyBOP (33 mg, 0.064 mmol, 1.0 eq), and DIEA (111 µL, 0.64 mmol, 10 eq) to yield the product. No 6Cl-HOBt was used. TFA deprotection yielded the product as a white solid. ^1^H-NMR (CD_3_OD, 500 MHz, rotamers) δ 7.36–7.33 (m, 2H), 7.32–7.23 (m, 7H), 7.21–7.13 (m, 4H), 6.85 (s, 1H), 6.41 (s, 2H), 6.21 (s, 2H), 4.72 (d, *J* = 17.3 Hz, 1H), 4.62 (d, *J* = 17.3 Hz, 1H), 4.64–4.57 (m, 2H), 4.44 (s, 2H), 4.42–4.35 (m, 5H), 4.29 (d, *J* = 16.0 Hz, 1H), 4.10 (dt, *J* = 13.0, 5.0 Hz, 1H), 3.73 (br s, 1H), 3.57 (d, *J* = 16.1 Hz, 1H), 3.46–3.35 (m, 2H), 3.28–3.26 (m, 1H), 3.26–3.15 (m, 6H), 3.15–3.07 (m, 2H), 2.80 (t, *J* = 6.0 Hz, 2H), 2.76–2.67 (m, 1H), 2.62 (dt, *J* = 16.5, 6.0 Hz, 1H), 2.24 (s, 6H), 2.22 (s, 6H), 2.04 (dt, *J* = 6.9, 5.0 Hz, 1H). HPLC retention time: 21.3 min. HREIMS *m*/*z* 470.2799 (calcd. for C30H35N3O2, 470.2802).

*Tert-butyl 7-((3,4-dihydroquinolin-1(2H)-yl)methyl)-3,4-dihydroisoquinoline-2(1H)-carboxylate* (**7x**). Compound **7x** was synthesized following General Procedure E from compound **3** (50 mg, 0.153 mmol, 1.0 eq), 1,2,3,4-tetrahydroquinoline (23 μL, 0.184 mmol, 1.2 eq), and K_2_CO_3_ (25 mg, 0.184 mmol, 1.2 eq). The reaction mixture was diluted with water. The aqueous layer was extracted with several portions of ethyl acetate. Combined organic layers were dried over MgSO_4,_ filtered, and concentrated under vacuum. Silica gel chromatography yielded a mixture of the desired product and 1,2,3,4-tetrahydroquinoline. The product was partitioned between ethyl acetate and 2 M NaOH. The aqueous layer was extracted with ethyl acetate. Some 1,2,3,4-tetrahydroquinoline remained. The mixture was resubmitted to reaction conditions with additional **3** (25 mg, 0.076, 0.5 eq) and K_2_CO_3_ (25 mg, 0.184 mmol, 1.2 eq). Silica gel chromatography yielded the desired product as a colorless oil (24 mg, 28%).^1^H-NMR (CDCl_3_, 500 MHz) δ 7.08 (s, 1H), 7.02 (s, 1H), 6.99 (t, *J* = 6.5 Hz, 2H), 6.59 (t, *J* = 7.3 Hz, 1H), 6.51 (d, *J* = 8.5 Hz, 1H), 4.55 (s, 2H), 4.44 (s, 2H), 3.65 (s, 2H), 3.36 (t, *J* = 5.7 Hz, 2H), 2.92–2.75 (m, 4H), 2.03 (p, *J* = 6.1 Hz, 2H), 1.50 (s, 9H).

*(S)-2-amino-1-(7-((3,4-dihydroquinolin-1(2H)-yl)methyl)-3,4-dihydroisoquinolin-2(1H)-yl)-3-(4-hydroxy-2,6-dimethylphenyl)propan-1-one* (**8x**). Following General Procedure F, intermediate **7x** (24 mg, 0.063 mmol, 1.0 eq) was deprotected to yield the amine intermediate. This intermediate was coupled to diBoc-DMT (27 mg, 0.067 mmol, 1.05 eq) in the presence of PyBOP (33 mg, 0.064 mmol, 1.0 eq), and DIEA (111 µL, 0.64 mmol, 10 eq) to yield the product. No 6Cl-HOBt was used. TFA deprotection yielded the product as a white solid. ^1^H-NMR (CD_3_OD, 500 MHz, rotamers) δ 7.09 (d, *J* = 7.9 Hz, 1H), 7.04 (d, *J* = 8.1 Hz, 2H), 7.01 (d, *J* = 7.9 Hz, 1H), 6.99–6.89 (m, 5H), 6.64–6.51 (m, 5H), 6.40 (s, 2H), 6.27 (s, 2H), 4.62 (d, *J* = 17.1 Hz, 1H), 4.58–4.53 (m, 2H), 4.51 (d, *J* = 17.0 Hz, 2H), 4.45 (s, 2H), 4.44 (s, 2H), 4.19 (d, *J* = 15.8 Hz, 1H), 3.93 (dt, *J* = 12.9, 5.4 Hz, 1H), 3.51–3.43 (m, 1H), 3.46 (d, *J* = 15.7 Hz, 1H), 3.40–3.34 (m, 4H), 3.26–3.19 (m, 3H), 3.11–3.05 (m, 2H), 2.81 (q, *J* = 5.9 Hz, 4H), 2.73 (t, *J* = 6.2 Hz, 2H), 2.69–2.62 (m, 1H), 2.54 (dt, *J* = 16.3, 6.1 Hz, 1H), 2.23 (s, 6H), 2.20 (s, 6H), 2.06–1.95 (m, 5H). HPLC retention time: 35.9 min. HREIMS *m*/*z* 470.2800 (calcd. for C30H35N3O2, 470.2802).

*Tert-butyl 7-(indolin-1-ylmethyl)-3,4-dihydroisoquinoline-2(1H)-carboxylate* (**7y**). Compound **7y** was synthesized following General Procedure E from compound **3** (50 mg, 0.153 mmol, 1.0 eq), indoline (21 μL, 0.184 mmol, 1.2 eq), and K_2_CO_3_ (25 mg, 0.184 mmol, 1.2 eq) to yield the product as a brown oil (37 mg, 66%). ^1^H-NMR (CDCl_3_, 500 MHz) δ 7.18 (d, *J* = 7.9 Hz, 1H), 7.15–7.08 (m, 3H), 7.07 (t, *J* = 7.6 Hz, 1H), 6.69 (t, *J* = 7.3 Hz, 1H), 6.52 (d, *J* = 7.8 Hz, 1H), 4.58 (s, 2H), 4.21 (s, 2H), 3.66 (s, 2H), 3.32 (t, *J* = 8.2 Hz, 2H), 2.99 (t, *J* = 8.3 Hz, 2H), 2.84 (t, *J* = 6.0 Hz, 2H), 1.51 (s, 9H).

*(S)-2-amino-3-(4-hydroxy-2,6-dimethylphenyl)-1-(7-(indolin-1-ylmethyl)-3,4-dihydroisoquinolin-2(1H)-yl)propan-1-one* (**8y**). Following General Procedure F, intermediate **7y** (37 mg, 0.102 mmol, 1.0 eq) was deprotected to yield the amine intermediate. This intermediate was coupled to diBoc-DMT (43 mg, 0.105 mmol, 1.05 eq) in the presence of PyBOP (52 mg, 0.100 mmol, 1.0 eq), and DIEA (174 µL, 1.0 mmol, 10 eq) to yield the product. No 6Cl-HOBt was used. TFA deprotection yielded the product as a white solid. ^1^H-NMR (CD_3_OD, 500 MHz, rotamers) δ 7.24–7.18 (m, 3H), 7.18–7.11 (m, 4H), 7.06 (d, *J* = 7.9 Hz, 1H), 7.03–6.94 (m, 4H), 6.89 (d, *J* = 8.5 Hz, 1H), 6.67 (s, 1H), 6.41 (s, 2H), 6.27 (s, 2H), 4.63 (d, *J* = 16.9 Hz, 1H), 4.60–4.56 (m, 2H), 4.53 (d, *J* = 17.2 Hz, 1H), 4.40–4.32 (m, 4H), 4.21 (d, *J* = 15.8 Hz, 1H), 3.89 (dt, *J* = 12.7, 5.5 Hz, 1H), 3.55–3.50 (m, 1H), 3.50–3.46 (m, 4H), 3.44 (d, *J* = 15.9 Hz, 1H), 3.28–3.17 (m, 3H), 3.13–3.06 (m, 2H), 2.99 (t, *J* = 7.6 Hz, 4H), 2.74 (t, *J* = 4.7 Hz, 2H), 2.72–2.65 (m, 1H), 2.56 (dt, *J* = 16.3, 6.0 Hz, 1H), 2.23 (s, 6H), 2.20 (s, 6H), 1.98 (dt, *J* = 16.2, 5.9 Hz, 1H). HPLC retention time: 30.1 min. HREIMS *m*/*z* 456.2644 (calcd. for C29H33N3O2, 456.2646).

*Tert-butyl 7-(isoindolin-2-ylmethyl)-3,4-dihydroisoquinoline-2(1H)-carboxylate* (**7z**). Compound **7z** was synthesized following General Procedure E from compound **3** (50 mg, 0.153 mmol, 1.0 eq), isoindoline HCl (29 mg, 0.184 mmol, 1.2 eq), and K_2_CO_3_ (25 mg, 0.184 mmol, 1.2 eq) to yield the product as an orange oil (22 mg, 39%). ^1^H-NMR (CDCl_3_, 500 MHz) δ 7.29 (s, 1H), 7.21 (d, *J* = 7.8 Hz, 1H), 7.18 (s, 4H), 7.12 (d, *J* = 7.6 Hz, 1H), 4.58 (s, 2H), 3.93 (s, 4H), 3.88 (s, 2H), 3.65 (t, *J* = 8.2 Hz, 2H), 2.84 (t, *J* = 6.2 Hz, 2H), 1.49 (s, 9H).

*(S)-2-amino-3-(4-hydroxy-2,6-dimethylphenyl)-1-(7-(isoindolin-2-ylmethyl)-3,4-dihydroisoquinolin-2(1H)-yl)propan-1-one* (**8z**). Following General Procedure F, intermediate **7z** (22 mg, 0.060 mmol, 1.0 eq) was deprotected to yield the amine intermediate. This intermediate was coupled to diBoc-DMT (26 mg, 0.063 mmol, 1.05 eq) in the presence of PyBOP (31 mg, 0.060 mmol, 1.0 eq), and DIEA (105 µL, 0.600 mmol, 10 eq) to yield the product. No 6Cl-HOBt was used. TFA deprotection yielded the product as a white solid. ^1^H-NMR (CD_3_OD, 500 MHz, rotamers) δ 7.41–7.38 (m, 8H), 7.37–7.34 (m, 2H), 7.30 (dd, *J* = 7.9, 1.8 Hz, 1H), 7.19 (dd, *J* = 7.9, 3.5 Hz, 1H), 7.16 (dd, *J* = 8.0, 3.7 Hz, 1H), 6.86 (s, 1H), 6.42 (s, 2H), 6.24 (s, 2H), 4.72 (d, *J* = 17.2 Hz, 1H), 4.70–4.64 (m, 8H), 4.61 (d, *J* = 15.3 Hz, 1H), 4.65–4.57 (m, 2H), 4.56 (s, 2H), 4.53 (d, *J* = 10.7 Hz, 2H), 4.29 (d, *J* = 16.0 Hz, 1H), 4.09 (dt, *J* = 13.2, 4.9 Hz, 1H), 3.57 (d, *J* = 15.9 Hz, 1H), 3.44–3.37 (m, 1H), 3.29–3.25 (m, 1H), 3.24 (q, *J* = 13.0 Hz, 2H), 3.15–3.07 (m, 2H), 2.80 (t, *J* = 4.3 Hz, 2H), 2.75–2.67 (m, 1H), 2.61 (dt, *J* = 16.2, 5.6 Hz, 1H), 2.25 (s, 6H), 2.23 (s, 6H), 2.07–2.00 (m, 1H). HPLC retention time: 20.1 min. HREIMS *m*/*z* 456.2645 (calcd. for C29H33N3O2, 456.2646).

### 4.2. Pharmacology

The pharmacological methods used were the same as those previously described [14], with any changes noted below. Unless otherwise noted, all tissue culture reagents and radiolabeled ligands were purchased from commercial sources. Cell lines were provided by Professor Lawrence Toll [34].

#### 4.2.1. Cell Lines and Membrane Preparations

Membranes prepared from transfected Chinese Hamster Ovary cells stably expressing human KOR, human MOR, or human DOR were used for all assays. Cells were grown to confluence at 37 °C in 5% CO_2_ in 1:1 DMEM:F12 media with 10% *v*/*v* fetal bovine serum and 5% *v*/*v* penicillin/streptomycin. Membranes were prepared by washing confluent cells three times with ice cold phosphate-buffered saline (0.9% NaCl, 0.61 mM Na_2_HPO_4_, 0.38 mM KH_2_PO_4_, pH 7.4). Cells were detached from the plates by incubation in warm harvesting buffer (20 mM HEPES, 150 mM NaCl, 0.68 mM EDTA, pH 7.4) and pelleted by centrifugation at 200× *g* for 3 min. The cell pellet was suspended in ice-cold 50 mM Tris-HCl buffer, pH 7.4 and homogenized with a Tissue Tearor (Biospec Products, Inc, distributed by Cole-Parmer, Vernon Hills, IL, USA) for 20 s at setting 4. The homogenate was centrifuged at 20,000× *g* for 20 min at 4 °C, and the pellet was rehomogenized in 50 mM Tris-HCl pH 7.4 with a Tissue Tearor for 10 s at setting 2, followed by recentrifugation. The final pellet was resuspended in 50 mM Tris-HCl pH 7.4 and frozen in aliquots at −80 °C. Protein concentration was determined via Pierce BCA protein assay kit using bovine serum albumin as the standard.

#### 4.2.2. Binding Affinity

Binding affinities for all test compounds at KOR, MOR, and DOR were determined by competitive displacement of [^3^H]-diprenorphine as previously reported [35,36,37,38]. In a 96-well plate format, cell membranes (5–10 µg of protein) and [^3^H]-diprenorphine (0.2 nM) were incubated in Tris-HCl buffer (50 mM, pH 7.4) with various concentrations of test compound at 25 °C for 1 h, allowing the mixture to reach equilibrium. Nonspecific binding was determined using the opioid antagonist naloxone (10 µM), and total binding was determined using vehicle in the absence of competitive ligand. After incubation, membranes were filtered through Whatman GF/C 1.2 micron glass fiber filters and washed with 50 mM Tris-HCl buffer. The radioactivity remaining on the filters was then quantified by liquid scintillation counting after saturation with EcoLume liquid scintillation cocktail in a Microbeta 2450 (Perkin-Elmer, Waltham, MA, USA). Binding affinity (K_i_) values were calculated using the Cheng–Prusoff equation via nonlinear regression analysis using GraphPad Prism software from at least three separate binding assays performed in duplicate.

#### 4.2.3. Stimulation of [^35^S]-GTPγS Binding

Agonist stimulation of KOR, MOR, and DOR by all test compounds was determined by [^35^S]-guanosine 5′-*O*-[γ-thio]triphosphate ([^35^S]-GTPγS) binding assays as previously reported [35,36,37,38]. In a 96-well plate format, membranes from cells expressing opioid receptors as described above (10 µg of protein), [^35^S]-GTPγS (0.1 nM), and guanosine diphosphate (30 µM) were incubated in GTPγS buffer (50 mM Tris-HCl, 100 mM NaCl, 5 mM MgCl_2_, 1 mM EDTA, pH 7.4) with various concentrations of test compound at 25 °C for 1 h. Basal stimulation was determined by incubation in the absence of any ligand. After incubation, membranes were filtered through Whatman GF/C 1.2 micron glass fiber filters and washed with GTPγS buffer with no EDTA. The radioactivity remaining on the filters was then quantified by liquid scintillation counting after saturation with EcoLume liquid scintillation cocktail in a Perkin-Elmer Microbeta 2450. Data are reported as percent stimulation compared to the effects of 10 µM standard agonists—U69,593 (KOR), DAMGO (MOR), or DPDPE (DOR). Percent stimulation and EC_50_ values were determined via nonlinear regression analysis using GraphPad Prism software from at least three separate assays performed in duplicate. Efficacy is expressed as percent stimulation relative to standard agonists.

## Figures and Tables

**Table 1:** Substituted 7-benzyl pendants on the Dmt-Tiq scaffold. ^1^

Compound	R	Ki (nM)			EC_50_ (nM)			% Stimulation		
		KOR	MOR	DOR	KOR	MOR	DOR	KOR	MOR	DOR
**4c**	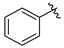	2.3 (0.3)	5.3 (0.5)	2.2 (0.7)	97 (24)	68 (15)	5.7 (2.0)	82 (6)	39 (4)	20 (3)
**8a**	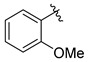	3.1 (0.9)	3.7 (0.6)	2.7 (0.7)	130 (41)	92 (26)	dns *	72 (11)	37 (8)	dns *
**8b**	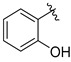	0.32 (0.01)	1.2 (0.1)	2.5 (0.3)	11 (1)	43 (13)	dns *	89 (6)	60 (5)	dns *
**8c**	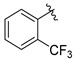	2.6 (0.6)	4.3 (1.3)	4.9 (2)	173 (55)	53 (11)	342 (32)	81 (11)	76 (6)	24 (3)
**8d**	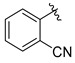	0.5 (0.1)	6.8 (0.2)	3.6 (0.8)	3.7 (0.8)	664 (515)	dns	80 (9)	31 (9)	dns
**8e**	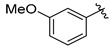	3.0 (0.9)	2.7 (0.6)	1.7 (0.4)	148 (38)	24 (3)	dns	74 (4)	48 (6)	dns
**8f**	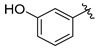	0.8 (0.2)	0.6 (0.2)	1.4 (0.1)	148 (53)	18 (3)	dns *	83 (5)	81 (9)	dns *
**8g**	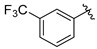	5.4 (1.1)	4.1 (1.1)	1.6 (0.5)	319 (115)	205 (96)	dns	68 (8)	45 (3)	dns
**8h**	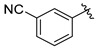	5.9 (0.9)	3.4 (1.3)	1.4 (0.2)	287 (61)	53 (6)	dns	60 (8)	29 (3)	dns
**8i**	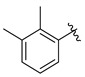	5.8 (1.2)	5.2 (1.4)	3.6 (0.6)	1030 (50)	380 (187)	dns *	54 (9)	34 (3)	dns *

^1^ Binding affinity (K_i_) values determined by competitive displacement of [^3^H]-diprenorphine in membrane preparations from CHO cells expressing human KOR, MOR, or DOR. Potency (EC_50_) and efficacy values determined by [^35^S]-GTPγS binding in the same membrane preparations. Efficacy expressed as percent stimulation versus standard agonists—U69,593 (KOR), DAMGO (MOR), or DPDPE (DOR). All values expressed as mean (SEM) of three or more separate assays run in duplicate unless otherwise noted. * *n* = 2; dns = does not stimulate, average maximal stimulation <10% at concentrations up to 10 µM.

**Table 2:** Pyridyl 7-position pendants on the Dmt-Tiq scaffold. ^1^

Compound	R	Ki (nM)			EC_50_ (nM)			% Stimulation		
		KOR	MOR	DOR	KOR	MOR	DOR	KOR	MOR	DOR
**8j**	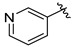	56 (0.9)	5.4 (0.3)	1.5 (0.2)	988 (277)	145 (30)	dns	40 (7)	23 (1)	dns
**8k**	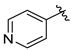	44 (12)	42 (18)	2.6 (1.0)	1180 (287)	179 (17)	dns	44 (9)	28 (1)	dns

^1^ Binding affinity (K_i_) values determined by competitive displacement of [^3^H]-diprenorphine in membrane preparations from CHO cells expressing human KOR, MOR, or DOR. Potency (EC_50_) and efficacy values determined by [^35^S]-GTPγS binding in the same membrane preparations. Efficacy expressed as percent stimulation versus standard agonists—U69,593 (KOR), DAMGO (MOR), or DPDPE (DOR). All values expressed as mean (SEM) of three or more separate assays run in duplicate unless otherwise noted. dns = does not stimulate, average maximal stimulation <10% at concentrations up to 10 µM.

**Table 3:** Aliphatic 7-position pendants on the Dmt-Tiq scaffold. ^1^

Compound	R	Ki (nM)			EC_50_ (nM)			% Stimulation		
		KOR	MOR	DOR	KOR	MOR	DOR	KOR	MOR	DOR
**8l**	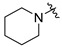	29 (11)	4.7 (0.9)	87 (15)	dns	845 (97)	dns	dns	34 (5)	dns
**8m**	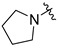	69 (3)	17 (11)	175 (9)	dns	639* (162)	dns *	dns	40 * (22)	dns *
**8n**	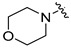	43 (17)	39 (27)	5.6 (1.6)	dns	860 (200)	dns	dns	38 (14)	dns

^1^ Binding affinity (K_i_) values determined by competitive displacement of [^3^H]-diprenorphine in membrane preparations from CHO cells expressing human KOR, MOR, or DOR. Potency (EC_50_) and efficacy values determined by [^35^S]-GTPγS binding in the same membrane preparations. Efficacy expressed as percent stimulation versus standard agonists—U69,593 (KOR), DAMGO (MOR), or DPDPE (DOR). All values expressed as mean (SEM) of three or more separate assays run in duplicate unless otherwise noted. * *n* = 2; dns = does not stimulate, average maximal stimulation <10% at concentrations up to 10 µM.

**Table 4:** Naphthyl 7-position pendants on the Dmt-Tiq scaffold. ^1^

Compound	R	Ki (nM)			EC_50_ (nM)			% Stimulation		
		KOR	MOR	DOR	KOR	MOR	DOR	KOR	MOR	DOR
**8o**	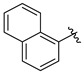	4.7 (0.5)	5.2 (1.0)	3.2 (0.6)	349 (112)	132 (76)	dns	83 (14)	59 (1)	dns
**8p**	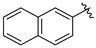	142 (23)	5.8 (1.6)	3.5 (0.7)	dns	176 (59)	dns	dns	51 (9)	dns

^1^ Binding affinity (K_i_) values determined by competitive displacement of [^3^H]-diprenorphine in membrane preparations from CHO cells expressing human KOR, MOR, or DOR. Potency (EC_50_) and efficacy values determined by [^35^S]-GTPγS binding in the same membrane preparations. Efficacy expressed as percent stimulation versus standard agonists—U69,593 (KOR), DAMGO (MOR), or DPDPE (DOR). All values expressed as mean (SEM) of three or more separate assays run in duplicate unless otherwise noted. dns = does not stimulate, average maximal stimulation <10% at concentrations up to 10 µM.

**Table 5:** Nitrogen scan of 7-position 1-naphthyl pendant on the Dmt-Tiq scaffold. ^1^

Compound	R	Ki (nM)			EC_50_ (nM)			% Stimulation		
		KOR	MOR	DOR	KOR	MOR	DOR	KOR	MOR	DOR
**8q**	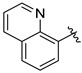	19 (2)	1.4 (0.3)	0.46 (0.08)	258 (16)	24 (4)	1.2 (0.8)	25 (3)	63 (8)	23 (4)
**8r**	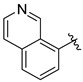	2.7 (0.7)	1.8 (0.3)	0.69 (0.24)	65 (19)	24 (6)	dns *	42 (15)	36 (5)	dns *
**8s**	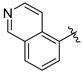	10 (1)	9.7 (1.4)	1.2 (0.2)	609 (226)	43 (21)	dns	24 (2)	28 (8)	dns
**8t**	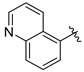	1.5 (0.5)	4.6 (0.5)	1.2 (0.4)	58 (11)	54 (15)	dns *	69 (1)	31 (6)	dns *
**8u**	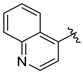	8.6 (0.4)	6.6 (1.0)	0.79 (0.25)	dns	54 (19)	dns *	dns	45 (12)	dns *
**8v**	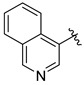	0.73 (0.03)	1.4 (0.2)	0.30 (0.04)	14 (1)	12 (3)	19 (10)	71 (16)	81 (7)	39 (7)

^1^ Binding affinity (K_i_) values determined by competitive displacement of [^3^H]-diprenorphine in membrane preparations from CHO cells expressing human KOR, MOR, or DOR. Potency (EC_50_) and efficacy values determined by [^35^S]-GTPγS binding in the same membrane preparations. Efficacy expressed as percent stimulation versus standard agonists—U69,593 (KOR), DAMGO (MOR), or DPDPE (DOR). All values expressed as mean (SEM) of three or more separate assays run in duplicate unless otherwise noted. * *n* = 2; dns = does not stimulate, average maximal stimulation <10% at concentrations up to 10 µM.

**Table 6:** Bicyclic aliphatic 7-position pendants on the Dmt-Tiq scaffold. ^1^

Compound	R	Ki (nM)			EC_50_ (nM)			% Stimulation		
		KOR	MOR	DOR	KOR	MOR	DOR	KOR	MOR	DOR
**8w**	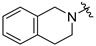	42 (8)	0.6 (0.1)	2.3 (0.6)	dns	8.2 (1.0)	dns *	dns	85 (9)	dns *
**8x**	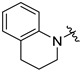	7.1 (1.4)	7.2 (1.6)	6.6 (1.0)	375 (109)	73 (22)	dns *	67 (3)	65 (10)	dns *
**8y**	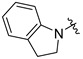	2.6 (0.6)	2.2 (0.6)	1.7 (0.5)	106 (20)	42 (10)	dns *	66 (8)	65 (5)	dns *
**8z**	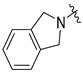	65 (23)	1.5 (0.2)	1.3 (0.4)	dns	27 (0.3)	dns *	dns	63 (10)	dns *

^1^ Binding affinity (K_i_) values determined by competitive displacement of [^3^H]-diprenorphine in membrane preparations from CHO cells expressing human KOR, MOR, or DOR. Potency (EC_50_) and efficacy values determined by [^35^S]-GTPγS binding in the same membrane preparations. Efficacy expressed as percent stimulation versus standard agonists—U69,593 (KOR), DAMGO (MOR), or DPDPE (DOR). All values expressed as mean (SEM) of three or more separate assays run in duplicate unless otherwise noted. * *n* = 2; dns = does not stimulate, average maximal stimulation <10% at concentrations up to 10 µM.
